# Supplementary material for: Changes in Caprine Milk Fat Globule Membrane Proteins after Heat Treatment Using a Label-Free Proteomics Technique
Source: Foods. 2022 Sep 5;11(17):2705. doi: 10.3390/foods11172705 (PMC9455663; doi:10.3390/foods11172705)
Supplement: Supplementary file 1 [file foods-11-02705-s001.zip › Table S1.pdf]

UniProt IDs	Gene	Protein	RAW_1	RAW_2	UP_1	UP_2	UHT_1	UHT_2	SP_1	SP_2
A0A452F2M2	CCT8	Chaperor	26.84	26.58	18.6	18.94	NaN	NaN	20.7	19.78
A0A452DUP3	RPN1	Dolichyl	29.58	29.75	18.81	19.24	NaN	18.76	25.75	25.39
A0A452ECC5	RPL4	Ribos_L4	28.33	27.88	18.83	19.97	NaN	NaN	NaN	NaN
A0A452DNT7	STX19	Syntaxir	23.37	23.16	19.35	NaN	20.69	20.4	21.08	20.9
A0A452EA76	RPSA	40S ribo	29.05	28.99	19.4	19.54	NaN	NaN	21.15	21.25
A0A452FUU6	PSME2	Proteaso	NaN	22.06	19.71	NaN	NaN	NaN	NaN	NaN
A0A452FNY6	TMEM8A	Transmem	22.19	21.62	19.72	NaN	19.75	20.6	21.44	21.9
A0A452DZN3	TMEM30B	Cell cyc	25.9	25.52	19.86	20.07	20.16	NaN	22.38	22.51
A0A452EYA9	SELENBP	Selenium	27.23	27.37	19.92	19.94	20.78	21.24	23.68	25.41
A0A452FXG0	N/A	F-actin-	23.71	23.6	20.03	20.07	NaN	NaN	21.09	21.4
A0A452FAR2	LMAN2	Lectin,	27.75	27.36	20.18	NaN	NaN	NaN	21.54	20.29
A0A452ECC6	PHB	Prohibit	24.39	24.72	20.26	20.51	NaN	20.34	21.39	21.08
A0A452E1M5	CAPN5	Calpain	23.67	23.3	20.29	21.42	21.84	22.08	23.43	23
A0A452FRD9	EPCAM	Epitheli	26.13	25.93	20.31	20.84	21.47	22.44	24.46	24.84
A0A452EWG1	COX5B	Cytochro	23.85	23.06	20.32	NaN	NaN	NaN	NaN	NaN
A0A452EWR7	PSMA3	Proteaso	25.58	25.81	20.34	20.18	NaN	NaN	21.59	22.25
A0A452ENI2	STX7	Syntaxir	22.26	21.61	20.43	20.13	NaN	NaN	22.19	21.88
A0A452FIL3	NPC1	SSD doma	23.85	24.15	20.49	20.72	21.76	22.04	22.79	23.3
A0A452DVV1	LAP3	Leucine	28.08	27.88	20.51	20.26	19.23	19.38	26.79	26.06
A0A452FHF8	PFDN6	Uncharac	23.86	22.16	20.55	NaN	NaN	NaN	21.35	NaN
A0A452EVI7	RPS3A	40S ribo	28.81	27.57	20.56	22.1	19.98	20.05	20.73	20.1
A0A452E3X6	ADGRE5	Adhesior	27.62	27.82	20.59	20.04	NaN	19.9	28.68	28.62
A0A452E2B3	PRSS23	Serine p	22.47	22.34	20.63	20.76	21.17	NaN	21.87	21.57
A0A452G0Z2	SEMA7A	Semaphor	28	27.8	20.64	22.18	25.05	24.34	26.88	26.92
A0A452EX47	N/A	COX6C do	23.47	23.67	20.68	20.47	20.61	20.84	NaN	NaN
A0A452FQ19	C4BPA	Uncharac	28.4	28.35	20.71	21.66	20.94	21.64	25.39	25.58
A0A452DRZ8	AKR1A1	Aldo-ket	28.09	26.8	20.72	NaN	20.1	19.62	23.63	23.71
A0A452EQZ4	MDH1	Malate c	25.76	25.75	20.73	20.77	NaN	20.66	23.73	24.26
A0A452FE61	EMC7	ER membr	23.84	23.84	20.78	21.15	NaN	NaN	NaN	NaN
A0A452E1R9	PRDX1	Thioredc	27.75	27.3	20.84	22.34	21.86	22.59	23.39	23.47
A0A452F2B2	GNAO1	G protei	27.29	27.06	20.85	NaN	24.16	23.72	25.41	26.06
A0A452E831	GNAQ	G protei	26.56	26.54	20.87	NaN	23.37	22.03	24.08	24.43
A0A452FTU7	FLOT1	Flotilli	27.38	26.7	20.87	22.32	20.51	NaN	25.18	24.63
A0A452EDW0	PBXIP1	PBX home	21.22	21.86	20.89	NaN	NaN	NaN	NaN	NaN
A0A452E2H1	ATP5PO	Uncharac	23.37	23.34	20.9	21.2	NaN	NaN	NaN	NaN
A0A452ETE8	CCT6A	Uncharac	26.27	26.61	20.9	21.12	NaN	NaN	22.09	21.9
A0A452FYA6	TMPRSS2	Transmem	26.47	25.98	20.97	21.42	NaN	NaN	24.82	24.49
A0A452ESM1	DLST	Dihydrol	24.08	23.85	21.01	21.46	NaN	21.02	NaN	22.12
A0A452FS20	PYD and	PYD and	22.85	22.66	21.03	21.16	NaN	NaN	NaN	22.24
A0A452E9Y4	ANP32A	Uncharac	21.48	NaN	21.07	21.43	NaN	NaN	NaN	21.56
A0A452G117	KRT10	Keratin	22.78	22.77	21.08	20.86	NaN	NaN	NaN	NaN
A7UAC5	N/A	Major pr	22.15	21.78	21.09	21.03	22.91	22.87	22.36	22.18
A0A452FWD9	DLD	Dihydrol	23.55	23.03	21.09	21.23	NaN	NaN	NaN	21.62
A0A452DWG5	SYNGR2	Synaptog	25.23	24.84	21.17	20.98	NaN	NaN	23.72	23.79
A0A452FWB2	PNPLA2	PNPLA dc	22.57	22.76	21.17	21.4	22.71	22.56	21.97	22.69
A0A452DRV9	PSAP	Prosapos	NaN	NaN	21.25	NaN	NaN	NaN	25.36	25.05
A0A452EWL1	UQCRC2	Ubiquinc	26.46	26.37	21.25	NaN	NaN	NaN	23	23.37
A0A452EK14	LHFPL2	LHFPL te	23.91	23.88	21.26	21.54	23.15	22.85	24.27	24.11
A0A452E803	LOC1065	Uncharac	25.37	25.04	21.3	21.24	NaN	19.38	21.21	20.79

A0A452ESU9	LOC1021	Uncharac	24.3	24.18	21.3	NaN	NaN	NaN	22.58	22.43
A0A452F261	TCN1	Transcol	22.07	NaN	21.34	21.83	NaN	NaN	22.33	21.99
A0A452EC41	EIF4A2	Eukaryot	24.78	25.4	21.35	21.19	NaN	NaN	22.7	22.27
A0A452E0X4	ERAP1	Aminopep	24.86	25.76	21.35	21.49	23.24	23.17	22.77	23.23
A0A452DW73	ACO2	Aconitat	24.69	23.93	21.38	22.02	21.6	NaN	NaN	NaN
A0A452DZT1	N/A	40S ribo	25.16	24.86	21.4	NaN	NaN	NaN	NaN	NaN
A0A452F283	SLC3A2	Solute c	26.84	26.53	21.41	23.64	24.18	23.7	25.9	25.59
A0A452FI53	SLC6A8	Transpor	25.66	25.19	21.42	NaN	21.83	22.61	23.21	24.24
A0A452FUQ6	SH3GLB2	SH3 doma	25.75	25.59	21.43	22.08	20.7	20.83	23.62	23.9
A0A452ELE7	C2	Compleme	21.02	NaN	21.48	21.01	25.24	25.09	23.27	22.97
A0A452FQU3	ITIH1	Inter-al	NaN	NaN	21.48	21.23	21.71	22.04	NaN	NaN
A0A452E5S8	NCL	Uncharac	NaN	NaN	21.5	21.99	NaN	NaN	NaN	NaN
A0A452F9H0	LOC1021	WAP doma	20.45	19.87	21.5	21.95	21.99	21.49	NaN	20.5
A0A452F0X4	ATP2B2	Calcium-	28.09	27.93	21.5	21.27	24.69	25.26	26.68	26.63
A0A452E2H5	PDZK1IP	PDZK1 ir	22.75	22.59	21.53	21.95	NaN	20.88	21.85	21.89
A0A452FF98	LIPE	Lipase E	24.93	25	21.58	NaN	23.63	23.65	24.41	24.09
A0A452ENU2	G6PD	Glucose-	25.27	24.91	21.59	21.9	NaN	NaN	NaN	NaN
A0A452FE95	RPL11	Uncharac	26.75	26.66	21.59	22.63	NaN	NaN	22.06	22.38
A0A452F756	PCYOX1	Prenylcy	25.4	25.98	21.66	NaN	NaN	NaN	24.27	23.87
A0A452EL72	MLEC	Malectir	26.97	26.88	21.67	NaN	NaN	NaN	22.43	23.58
A0A452FDD0	N/A	Uncharac	NaN	NaN	21.68	21.36	NaN	NaN	NaN	NaN
A0A452FND2	SLC29A1	Solute c	25.39	25.28	21.69	NaN	22.63	23.02	24.39	24.31
A0A452EZB5	CD55	Uncharac	23.09	23.46	21.69	NaN	23.24	23.37	25.26	25.08
A0A452FGC9	CAPG	Capping	26.38	26.34	21.7	21.36	21.3	NaN	23.37	22.26
A0A452E7X0	SH3BGRL	SH3 doma	NaN	NaN	21.73	20.99	NaN	NaN	NaN	NaN
A0A452FTQ1	NAPA	NSF atta	28.95	28.85	21.74	21.49	23.81	24	27.29	27.37
A0A452EU71	TMED5	Transmen	NaN	24.08	21.76	NaN	NaN	21.82	NaN	NaN
A0A452END8	TMED3	Transmen	25.07	25.1	21.77	22.8	NaN	NaN	NaN	NaN
A0A452DTK8	AGRN	Agrin	21.92	22.02	21.8	NaN	NaN	NaN	21.74	21.93
A0A452F2X0	SNX5	Sorting	22.13	21.97	21.8	NaN	NaN	NaN	22.42	22.47
A0A452DM06	GOLGA7	Golgin A	26.32	25.12	21.81	22.04	22.58	24.29	24.55	24.24
A0A452EMT1	RAB3D	RAB3D, m	28.22	27.86	21.83	21.97	22.97	23.79	25.64	25.53
A0A452FXH3	PSMD6	Proteasc	24.35	25.71	21.84	NaN	NaN	NaN	NaN	NaN
A0A452DKP9	SDF2L1	Stromal	24.04	NaN	21.86	22.67	NaN	NaN	NaN	NaN
A0A452FA18	TMC5	Transmen	27.58	27.42	21.87	22.58	23.89	23.61	25.48	25.48
A0A452EHH6	SMIM1	Small ir	22.83	NaN	21.88	NaN	NaN	NaN	21.28	21.66
A0A452FZ04	APOH	Apolipop	25	25.65	21.89	22.43	21.88	22.73	24.21	23.26
A0A452FTU1	SELENOS	Uncharac	22.89	NaN	21.9	21.46	NaN	NaN	NaN	NaN
A0A452DQS6	RAB21	RAB21, m	27.03	26.65	21.91	23.77	24.59	24.77	26.18	26.18
A0A452DVV9	LRRC59	Leucine	25.32	25.98	21.94	22.83	NaN	NaN	22.68	NaN
A0A452G9R5	FAM20A	FAM20A g	24.29	24.18	21.98	NaN	NaN	21.68	25.24	24.1
A0A452F322	LOC1021	Peptidas	NaN	NaN	21.98	22.03	NaN	NaN	NaN	NaN
G3EHG5	N/A	Perilipi	NaN	22.56	21.98	NaN	NaN	NaN	23.83	23.69
A0A452EIR1	EEF2	Tr-type	29.14	29.18	21.99	NaN	24.51	23.59	26.96	26.53
A0A452FPR6	PRDX4	Peroxire	26.58	26.5	21.99	21.88	22.47	NaN	25.39	24.72
A0A452FU98	ANGPTL4	Angiopoi	22.18	22.35	22.03	22.33	NaN	NaN	NaN	NaN
A0A452E197	ITGAV	Integrir	23.81	24.25	22.04	21.91	21.38	NaN	24.87	23.98
A0A452EY83	DNAJB2	DnaJ hea	24.99	24.79	22.04	22.15	NaN	NaN	22.96	22.74
A0A452EQZ3	APH1A	Aph-1 hc	24.84	24.22	22.06	22.18	NaN	NaN	23.57	23.48
A0A452FQZ4	QSOX1	Sulfhydr	24.27	24.39	22.06	22.16	26.86	27.03	25.47	26.11

A0A452EP01	MGST1	Microson	25.34	25.62	22.07	NaN	NaN	NaN	NaN	NaN
A0A452FT24	RPS4X	40S ribo	28.37	28.15	22.07	22.13	20.79	NaN	20.27	20.14
A0A452ET76	SNRPD2	Small nu	NaN	NaN	22.07	21.77	NaN	NaN	NaN	NaN
A0A452DWG6	MYADM	Myeloid	25.38	25.2	22.11	21.93	22.57	22.93	24.7	24.83
A0A452FNI4	FIS1	Mitochor	25.02	25.44	22.12	21.92	21.69	22.14	25.27	25.01
A0A452FQ03	RRBP1	Ribosome	26.5	26.09	22.12	22.4	NaN	22.02	23.43	22.61
A0A452E2I5	IQGAP1	IQ motif	24.63	25.19	22.12	22.39	NaN	NaN	21.95	22.05
A0A452E5R9	SLC9A3R	Na(+)/H(	24.63	24.25	22.12	22.04	NaN	20.81	22.87	23.09
A0A452DU53	CNP	2',3'-cy	25.38	25.13	22.12	21.83	23.74	23.71	25.16	24.93
A0A452DPN1	SHMT2	Serine h	22.32	22.06	22.13	NaN	22.04	21.71	NaN	NaN
A0A452G9G0	RPL38	Uncharac	24.48	24.47	22.13	22.78	NaN	NaN	22.36	NaN
A0A452EN49	HNRNPK	Uncharac	22.6	NaN	22.14	22.55	NaN	NaN	NaN	NaN
A0A452EEZ4	MOB1B	MOB kina	26.77	26.89	22.15	21.85	22.44	21.63	24.52	24.79
A0A452G6M6	LOC1021	Uncharac	24.24	23.92	22.16	21.35	NaN	NaN	NaN	21.04
B6RT17	RAB27A	RAB27A,	25.63	25.66	22.2	22.16	23.78	23.44	24.19	24.29
A0A452FNH6	EIF1B	Eukaryot	24.72	24.61	22.2	22.45	22.03	21.9	22.92	23.61
P00712	LALBA	Alpha-la	NaN	NaN	22.2	22.32	22.42	22.36	22.86	22.6
A5JSS3	NDUFA4	NADH deh	23.35	22.74	22.23	22.23	NaN	NaN	NaN	NaN
A0A452DTF9	CLEC3B	C-type l	23.55	23.49	22.24	22.3	21.89	21.77	24.02	24.06
A0A452FBP1	TMED4	Transmem	24.66	25.02	22.24	22.4	NaN	NaN	21.89	21.94
A0A452EGP3	TXN	Thioredo	27.82	28	22.26	23	22.51	NaN	26.31	23.97
A0A452E0Z2	GAPDHS	Glyceral	22.6	22.27	22.27	21.94	21.86	21.42	NaN	NaN
A0A452EP50	UBQLN1	Ubiquili	24.94	25.03	22.28	NaN	NaN	NaN	23.14	23.23
A0A452EGC4	GOT2	Aspartat	24.22	24.3	22.28	22.27	NaN	NaN	NaN	NaN
A0A452G990	AHCY	Adenosyl	27.94	27.93	22.28	22.84	22.75	NaN	24.62	24.66
A0A452DZZ5	LOC1021	SERPIN d	25.88	25.98	22.29	22.98	24.85	24.73	25.75	26
Q3LUG9	N/A	Mitochor	NaN	NaN	22.29	NaN	NaN	20.42	NaN	NaN
A0A452EZK6	RAB22A	RAB22A,	26.61	25.5	22.3	NaN	23.21	21.81	24.66	23.97
A0A452DRQ8	ERO1A	Endoplas	25.43	26.21	22.3	21.71	23.49	23.48	24.55	24.98
A0A452E9E9	CNPY2	Saposin	25.33	24.26	22.33	22.63	24.62	24.82	24.32	24.12
A0A452G6W6	NSFL1C	NSFL1 co	23.38	23.3	22.34	22.15	NaN	NaN	NaN	NaN
Q9TTY8	GSTP1	Glutathi	26.13	25.9	22.36	22.99	22.21	22.19	25.05	24.78
A0A452FKN5	ANKRD13	Ankyrin	26.71	25.71	22.38	NaN	24.18	NaN	NaN	21.32
A0A452FKK3	PFDN2	Uncharac	23.73	24.8	22.38	22.22	NaN	NaN	23.07	23
A0A452DRN1	CTSD	SNRPD1	NaN	NaN	22.39	NaN	NaN	NaN	23.31	23.25
A0A452ELN3	MSRA	Methioni	23.11	23.79	22.39	NaN	NaN	NaN	23.57	23.24
A0A452DKU0	RPS24	40S ribo	24.97	24.7	22.39	22.73	20.52	NaN	NaN	NaN
A0A452DKU9	VCL	Vinculin	23.32	22.92	22.4	NaN	24.14	23.61	23.29	23.48
A0A452FAB7	SARS	Seryl-tR	23.5	24.29	22.41	22.06	NaN	NaN	23.36	23.37
G1CQF2	HSP70.1	Heat sho	29.12	29.1	22.41	22.41	NaN	NaN	25.73	24.73
A0A452FRZ3	COX6B1	Cytochro	24.8	24.69	22.43	22.16	NaN	NaN	NaN	NaN
A0A452FQK4	ABHD14B	Abhydrol	24.56	24.27	22.44	21.37	NaN	NaN	NaN	23.32
A0A452EYS8	PHB2	Prohibit	26.05	26.76	22.44	21.55	NaN	NaN	NaN	22.1
A0A452FK27	HSPA13	Heat sho	23.94	24.5	22.45	22.59	NaN	NaN	25.6	25.3
A0A452G4S6	ELANE	Elastase	25.58	25.65	22.46	22.44	NaN	NaN	22.69	22.91
A0A452DT76	ATP11B	Phosphol	25.4	26.69	22.46	22.49	NaN	NaN	25.08	26.33
A0A452EW53	RCN2	Reticulo	24.33	23.31	22.47	23.16	NaN	NaN	NaN	22.51
A0A452F5Q3	STXBP2	Uncharac	27.44	27.4	22.47	21.54	24.01	24.26	25.79	25.52
A0A452EXD0	FXYD6	FXYD dom	23.28	23.34	22.48	21.93	21.62	21.27	22.83	22.58
A0A452ELX4	RPL15	Ribosoma	27.77	27.77	22.49	22.42	NaN	19.66	NaN	22.84

A0A452E8Q0	LGALS3	Galectir	21.73	22.27	22.49	22.2	20.94	NaN	NaN	NaN
A0A452EW49	LOC1021	Uncharac	NaN	NaN	22.5	22.81	23.13	23.09	22.79	22.99
A0A452EVP1	APOD	Apolipop	25.04	24.08	22.51	22.25	22.48	22.29	22.98	NaN
A0A452EJK8	P4HA1	Prolyl 4	22.45	22.62	22.51	22.57	NaN	NaN	NaN	NaN
A0A452EK81	N/A	Uncharac	22.4	22.36	22.52	23	NaN	NaN	23.05	22.94
A0A452G2Z3	CHMP1A	Charged	25.07	24.81	22.53	22.68	NaN	NaN	25.39	25.35
A0A452ELS1	LOC1021	SERPIN c	NaN	NaN	22.54	23.35	21.61	22.02	NaN	NaN
A0A452FJ07	LOC1021	SERPIN c	NaN	NaN	22.54	21.97	21.66	21.77	NaN	NaN
A0A452F9Y6	RPS5	Ribosoma	26.06	26.13	22.55	NaN	NaN	NaN	NaN	NaN
A0A452F2Z2	LOC1021	GLOBIN c	NaN	NaN	22.55	22.8	NaN	22.65	24.21	24.12
P80601	RIDA	2-iminob	NaN	NaN	22.56	22.13	21.13	NaN	21.65	NaN
A0A452EZU7	RHEB	Uncharac	28.52	28.48	22.56	22.75	23.41	23.68	25.58	25.42
A0A452E1K0	CMPK1	UMP-CMP	26.29	26.34	22.58	22.39	NaN	21.82	26.27	26.29
A0A452G373	RPL28	Ribosoma	27.23	26.48	22.58	23.48	23.15	NaN	NaN	21.79
A0A452FM11	TUBB4B	Tubulin	23.46	23.47	22.59	22.91	20.87	22.12	22.88	22.8
A0A452FRD3	CLINT1	Clathrir	24.31	24.21	22.59	22.45	22.04	21.86	23.37	23.39
S5U2L5	BPI	Bacteric	27.91	27.59	22.59	24.76	24.99	25.41	28.91	28.39
A0A452EE39	ITM2B	Integral	25.1	24.55	22.6	22.61	20.76	NaN	26.05	25.9
A0A452EWB0	MCEMP1	Uncharac	NaN	NaN	22.6	21.9	NaN	NaN	NaN	NaN
A0A452DRY1	UBL3	Ubiquiti	26.44	26.5	22.6	24.5	25.63	25.69	26.13	26.09
A0A452ELT4	PRTN3	Proteina	24.51	24.12	22.61	22.2	NaN	NaN	23.07	23.25
A0A452EUH4	BPI	Uncharac	25.44	25.48	22.62	22.4	NaN	NaN	26.44	26.38
A0A452FDR2	RPS8	40S ribo	28.01	27.77	22.66	22.77	19.37	19.17	21.86	20.12
A0A452DLC3	VAPA	VAMP ass	24.01	24.59	22.66	NaN	NaN	NaN	NaN	NaN
A0A452F525	SFTPD	C-type l	23.28	23.7	22.66	22.25	23.11	23.38	23.81	23.89
A0A452EL60	ACTR2	Actin-re	25.73	25.64	22.68	22.64	NaN	NaN	24.16	23.78
A0A452FDF6	MTDH	Metadher	23.04	23.56	22.69	22.65	NaN	NaN	NaN	NaN
A0A452F147	AUP1	AUP1 lip	26.77	26.47	22.69	NaN	24.24	24.05	25.78	25.5
A0A452G7E5	TG	Thyroglo	NaN	NaN	22.73	22.65	23.62	23.98	23.75	23.86
A0A452EEQ0	LOC1086	Uncharac	25.12	25.1	22.73	22.78	22.21	22.52	24.77	24.72
A0A452DJZ6	CNDP2	Carnosir	24.85	24.42	22.74	22.73	22.76	22.86	23.56	24.12
A0A452FEW9	BTF3	Transcri	23	23.11	22.74	22.82	NaN	NaN	NaN	NaN
A0A452EG05	PRKCSH	Protein	25.76	25.64	22.76	22.3	NaN	NaN	25.65	25.62
A0A452FY20	CAB39	Calcium	25.91	26.22	22.76	21.92	21.94	22.51	24.64	24.09
A0A452G137	GC	GC vitam	24.27	24.05	22.78	NaN	NaN	NaN	25.03	25.26
A0A452G6U3	RHOF	Ras homo	25.13	25.05	22.78	22.93	24.78	24.72	25.33	25.49
A0A452FWS6	N/A	Uncharac	27.38	26.09	22.78	NaN	22.72	21.68	28.12	27.83
A0A452FXM5	VDAC1	Voltage	26.03	26.81	22.81	21.98	NaN	NaN	NaN	NaN
A0A452GBG0	N/A	Ribosoma	26.64	26.57	22.81	23.13	NaN	20.44	22.35	22.26
A0A452EGN1	RHOG	Ras homo	25.94	26.04	22.82	23.37	24.58	24.44	25.92	25.7
A0A452G6D2	PGD	6-phosph	27.01	26.59	22.83	21.98	25.18	24.78	26.45	26.16
A0A452DTS3	ANKRD22	Ankyrin	25.53	25.8	22.85	22.57	24.71	24.84	25.31	25.06
A0A452EZJ3	MAL2	Mal, T c	26.58	26.42	22.87	NaN	NaN	21.85	24.52	24.37
A0A452DVL5	LOC1086	Uncharac	25.83	25.69	22.89	NaN	NaN	NaN	21.98	NaN
A0A452G5D5	RPL10A	Ribosoma	27.02	27.43	22.9	23.58	22.09	20.38	23.19	22.87
A0A452FJ15	NME2	Nucleosi	27.28	27	22.9	24.36	NaN	20.01	25.82	25.46
A0A452DN94	PITPNB	Phosphat	28.47	28.43	22.91	23.97	25.51	25.43	27.04	27.15
A0A452G0W0	RPL8	Ribosoma	27.07	26.82	22.92	23.38	20.97	NaN	22.18	22.67
A0A452FYF6	AK3	GTP:AMP	NaN	NaN	22.93	23.03	NaN	NaN	NaN	NaN
A5JST3	N/A	Translat	25.92	25.93	22.93	NaN	NaN	NaN	24.06	24.32

A0A452G8G7	SERBP1	SERPINE1	23.8	24.13	22.94	23.49	NaN	NaN	NaN	NaN
A0A452EB62	N/A	Uncharac	24.16	24.65	22.95	22.91	NaN	NaN	NaN	NaN
A0A452E8H3	LGALS3	Galectir	25.56	25.7	22.95	21.72	21.83	NaN	25.25	24.95
A0A452DW85	RPL7	Uncharac	27.65	27.58	22.96	23.06	NaN	NaN	NaN	22.55
A0A452FMD4	RAB13	Uncharac	25.49	25.28	22.96	23.06	24.02	23.9	24.94	24.79
A0A452FQK5	MIF	Macropha	24.88	25.24	22.97	23.25	22.09	22.69	24.17	24.01
A0A452FE54	IER3IP1	Immediat	23.69	25.67	23	23.12	NaN	NaN	22.96	23.07
A0A452FTV0	LOC1086	Annexin	25.14	25.19	23.03	23.55	23.38	23.61	24.69	24.85
A0A452FC08	PITPNA	Phosphat	26.37	26.28	23.03	23.22	24.01	24.01	25.6	25.61
A0A097C2Z0	RPLP0	60S acic	29.09	29.64	23.03	21.65	NaN	NaN	25.31	24.62
A0A452DXS5	ARF6	ADP ribc	26.53	26.71	23.04	23.69	24.57	24.69	25.47	25.53
A0A452EC63	BCL2L15	BCL2 lik	23.02	22.81	23.04	23.38	NaN	NaN	22.58	22.62
A0A452F9G8	RPL14	Ribosoma	27.45	27.43	23.04	22.88	21.96	22.42	22.55	22.32
A0A452FC02	SLC5A1	Uncharac	27.49	27.67	23.04	22.98	24.93	25.24	26.41	26.35
A0A452EX53	LOC1065	Serum am	22.32	22.65	23.06	23.75	22.59	22.78	22.91	22.86
A0A452FR38	RPL12	Uncharac	27.23	27.5	23.06	23.18	22.3	NaN	25.28	24.95
A0A452G3W0	OLFML3	Olfactom	24.96	25.15	23.06	22.89	23.18	23.21	24.79	24.58
A0A452DVX7	LOC1021	Peptidas	24.49	25.98	23.06	22.73	NaN	NaN	NaN	NaN
A0A452FPC1	HNRNPC	RRM doma	NaN	NaN	23.07	22.74	NaN	NaN	NaN	NaN
A0A452G0P1	NCF2	Neutroph	NaN	NaN	23.07	20.6	18.63	18.77	19.18	17.82
A0A452DP05	CHMP6	Charged	26.35	25.84	23.07	22.87	24.07	24.32	24.68	24.76
A0A452G6G9	ERLIN2	ER lipic	29.53	29.86	23.09	24.53	NaN	NaN	27.62	27.67
A0A452FMD5	GLIPR2	SCP doma	24.53	24.4	23.1	23.28	23	23.27	23.45	22.96
A0A452E1A4	PSME1	Proteasc	24.72	24.14	23.11	22.56	21.02	20.9	23.23	22.7
A0A452FNU3	LOC1021	Uncharac	27.13	27.38	23.11	22.56	NaN	NaN	23.52	23.7
A0A452FYL8	GNAI3	G protei	26.55	25.8	23.12	23.31	25.06	24.65	26.13	NaN
A0A452FIH6	MCFD2	Multiple	28.47	28.26	23.13	22.06	NaN	NaN	28.86	29.24
A0A452EEP5	MSN	Moesin	23.67	23.39	23.13	23.56	NaN	NaN	23.91	23.32
A0A452FM54	PARK7	Parkinsc	24.97	25.01	23.13	23.88	NaN	NaN	24.89	24.02
A0A452EMA0	PSMA7	Proteasc	25.04	25.69	23.13	23.17	NaN	NaN	22.85	22.64
A0A452FKI3	UGP2	UTP--glu	29.87	29.82	23.14	24.61	NaN	NaN	26.75	26.92
A0A452FZD1	RPL22	Ribosoma	26.96	26.85	23.15	23.42	NaN	NaN	22.73	NaN
A0A452DP35	N/A	L-lactat	23.71	23.6	23.15	22.78	22.89	22.77	23.57	23.53
A0A452E178	N/A	Histone	21.66	22.43	23.16	22.87	19.33	20.58	20.59	21.44
A0A452EFS9	RPL34	Ribosoma	26.55	26.62	23.17	23.1	21.42	NaN	22.97	23.24
A0A452ECV2	RPS14	Uncharac	27.65	27.64	23.18	23.53	NaN	NaN	22.09	23.86
A0A452E5B4	PRDX5	Peroxire	24.2	25.2	23.19	23.33	22.01	NaN	24.13	24.22
A0A452FWX5	N/A	Ig-like	25.16	25.44	23.2	23.47	24.51	24.89	24.14	23.99
A0A452FUR0	UBXN4	UBX doma	24.67	24.69	23.22	23.38	23.52	23.39	24.33	24.51
A0A452DRK5	HPX	Hemopexi	NaN	NaN	23.23	23.5	25.93	24.27	NaN	23.3
A0A452DNE9	LOC1021	Uncharac	23.04	22.78	23.24	23.35	24.16	23.49	24.67	23.43
A0A452F6M6	AP3B1	AP-3 com	NaN	NaN	23.25	22.79	23.6	23.29	22.67	NaN
A0A452FWS5	SLC39A7	Solute c	24.45	24.97	23.25	23.2	NaN	NaN	22.88	22.09
A0A452E238	LOC1021	Ribosoma	28.27	28.83	23.28	23.5	21.55	NaN	24.38	24.09
A0A452EVH6	ANXA6	Annexin	26.73	26.95	23.3	22.15	24.6	24.57	26.72	26.41
A0A452G3B2	SEC61B	Protein	24.02	23.93	23.31	23.08	20.88	NaN	22.38	22.38
A0A452GB27	RPS11	Ribosoma	27.89	25.52	23.32	23.36	NaN	NaN	NaN	NaN
A0A452EMY5	FGG	Fibrinog	28.83	28.82	23.32	23.09	25.4	25.03	27.89	27.82
A0A452F3L8	TUBA1B	Tubulin	25.76	25.16	23.33	24.03	22.84	22.92	24.68	24.64
A0A452ESM6	ARPC4	Actin-re	25.92	26.03	23.34	23.04	22.66	22.79	25.17	25.12

A0A452EAA6	N/A	Uncharac	26.44	26.38	23.34	23.38	NaN	NaN	NaN	NaN
A0A452E613	TALDO1	Transalc	22.45	21.94	23.35	NaN	22.33	21.52	23.78	23.87
A0A452DUS0	ACO1	Aconitas	28.35	28.2	23.36	NaN	23.58	23.39	25.41	25.79
A0A452DKT5	SNRPD3	Small nu	NaN	NaN	23.36	23.62	NaN	NaN	NaN	NaN
A0A452FUC4	PPBP	C-X-C mc	23.91	23.89	23.36	23.85	21.31	21.44	22.97	23.28
A0A452E7W7	LOC1086	C-type l	24.47	24.08	23.37	23.28	22.26	NaN	25.07	25.25
A0A452FMT2	LOC1086	S5 DRBM	28.46	28.57	23.37	23.28	NaN	NaN	22.27	20.75
A0A452E432	PROS1	Protein	22.49	22.67	23.37	24.01	23.22	NaN	24.66	24.49
A0A452FEY7	CHMP4A	Charged	25.08	25.32	23.37	23.38	NaN	NaN	24.84	23.69
A0A452EFS5	LSS	Terpene	28.66	28.64	23.37	21.86	26.3	26.48	27.83	27.78
A0A452EX45	LOC1021	Uncharac	28.47	28.1	23.39	24.02	25.17	24.75	25.69	27.13
A0A452EHX9	LOC1021	Cytochrc	24.53	25.03	23.4	23	NaN	NaN	22.84	NaN
A0A452ELY8	LOC1021	Ribosoma	28.35	28.18	23.41	23.7	NaN	NaN	NaN	23.36
A0A452EBH4	ATP5PB	ATP synt	26.83	25.57	23.41	23.2	NaN	NaN	21.41	NaN
A0A452G388	MOGS	Mannosyl	24.8	25.62	23.41	NaN	NaN	NaN	NaN	NaN
A0A452EP10	KRT71	Keratin	NaN	NaN	23.43	23.67	NaN	NaN	NaN	NaN
A0A452FUL8	GPX4	Uncharac	25.7	25.62	23.45	23.75	24.36	24.49	26.01	25.68
A0A452EJX2	RTN3	CCT4	26.53	26.72	23.49	23.52	22.83	22.79	24.83	24.99
A0A452EU40	RPL23	Uncharac	26.02	24.32	23.49	23.33	NaN	NaN	NaN	23.04
A0A452E7S2	RPL9	Uncharac	26.92	27.19	23.49	23.02	NaN	NaN	NaN	NaN
A0A452F9F5	N/A	Ig-like	23.02	22.57	23.49	23.98	24.33	24.61	NaN	23.29
A0A452FWU9	RPS16	Ribosoma	28.01	28.16	23.5	23.41	NaN	NaN	23.41	22.85
A0A452ECA8	ITGAL	VWFA dom	25.07	25.02	23.5	23.5	22.55	22.44	24.23	24.59
A0A452DZN6	PHIP	Pleckstr	23.48	23.24	23.52	23.95	24.98	24.89	23.88	23.87
A0A452EQT0	FCER1G	Fc fragn	NaN	NaN	23.52	24.07	NaN	NaN	NaN	NaN
A0A452DRM6	SNRPD1	Small nu	NaN	NaN	23.55	23.68	NaN	NaN	NaN	NaN
A0A452ELB1	SLC7A4	Solute c	28.39	27.99	23.55	23.57	25.04	25.36	27.63	26.36
A0A452GB32	ATP5F1D	ATP synt	24.05	24.06	23.57	23.64	NaN	NaN	23.87	23.94
A0A452F509	ATP5F1C	ATP synt	24.9	25.41	23.57	23.7	NaN	NaN	23.35	23.79
A0A452G9H1	EEF1B2	Uncharac	26.3	27.58	23.57	23.84	NaN	NaN	24.48	24.18
A0A452FSH3	CAPZA1	F-actin-	27.94	28.08	23.57	24.26	22.78	22.17	25.76	25.31
A0A452FID4	PDCD10	Uncharac	26.73	26.78	23.59	24.19	22.97	22.9	25.63	25.4
A0A452DL82	ANXA11	Annexin	24.81	24.82	23.6	23.67	23.73	23.63	25.64	25.28
A0A452FJ56	NPTN	Neuropla	27.45	27.49	23.6	23.41	24.12	23.86	26.73	26.53
G1DFP3	THBS1	Thrombos	23.18	23.18	23.62	23.41	22.55	23.19	24.57	24.44
A0A452G0K4	RRAS	RAS rela	28.4	28.36	23.62	NaN	26.2	26.28	26.88	26.53
A0A452E8D3	N/A	Ig-like	26.15	26.2	23.63	23.53	24.98	25.38	25.33	25.17
A0A452GAN5	SET	Uncharac	NaN	NaN	23.65	23.54	NaN	NaN	NaN	22.58
A0A452DTF4	BASP1	Brain ab	NaN	NaN	23.66	23.72	22.91	22.8	NaN	NaN
A0A452FSF6	NRAS	NRAS prc	26.38	26.6	23.68	NaN	24.46	NaN	26.32	25.52
A0A452E294	SOD2	Superoxi	24.56	24.54	23.69	23.35	NaN	NaN	NaN	NaN
A0A452F5L6	CD177	CD177 mc	24.16	24.12	23.69	23.73	23.85	24.14	25.84	25.23
A0A452E9T4	LOC1021	Uncharac	28.81	28.59	23.69	23.77	26.38	26.45	27.51	27.61
A0A452FTL9	VAPB	VAMP ass	24.66	24.98	23.7	23.03	NaN	NaN	22.53	22.54
A0A452DVS5	N/A	Protein	26.21	26.18	23.71	23.46	NaN	NaN	22.9	22.99
Q32643	MT-ATP8	ATP synt	24.28	23.76	23.71	23.5	NaN	NaN	NaN	NaN
A0A452EVW5	SELENOM	Selenopr	24.51	24.94	23.71	23.45	NaN	NaN	24.26	23.95
A0A452EHS0	RBMX	RRM doma	22.89	NaN	23.72	24.26	NaN	NaN	23.38	NaN
A0A452FB40	GLTP	Glycolip	26.37	26.24	23.72	23.55	24.53	24.94	26.31	26.26
A0A452EFK6	ATP6AP2	Uncharac	25.07	25.65	23.73	23.32	24.57	24.04	25.2	26.36

A0A0U5ADC8	N/A	Kappa ca	22.53	22.34	23.73	23.68	23.39	23.35	23.78	23.62
A0A452EYY1	ARPC5	Actin-re	24.18	23.35	23.75	23.5	NaN	NaN	24.82	25.68
A0A452EN63	FGB	Fibrinog	28.46	28.62	23.75	21.55	23.36	24.03	27.18	27.74
A0A452EHK2	NACA	NAC-A/B	25.06	25.43	23.75	23.34	21.86	NaN	22.9	23.56
A0A452F602	LOC1021	Uncharac	21.89	21.87	23.76	23.87	NaN	21.29	NaN	21.59
A0A452G2G1	S100A4	Protein	22.07	22.23	23.77	23.78	NaN	NaN	NaN	NaN
A0A452FZP6	ASRGL1	Asparagi	NaN	NaN	23.78	NaN	NaN	NaN	23.21	NaN
A0A452G5U6	LOC1021	Cytochro	25.47	24.65	23.79	24.23	NaN	NaN	23.44	NaN
A0A452FLH1	RAB7B	RAB7B, m	27.27	26.86	23.8	23.05	24.44	25.01	26.85	27.02
A0A452EB42	VASP	Vasodila	22.34	NaN	23.82	23.95	NaN	NaN	NaN	NaN
A0A452EAS2	LOC1086	SERPIN c	NaN	NaN	23.83	23.84	21.97	22.42	NaN	NaN
A0A452FU62	RPS17	Uncharac	24.03	25.12	23.86	22.99	NaN	NaN	NaN	NaN
A0A452EWU7	MYDGF	Myeloid	24.31	24.37	23.86	23.83	22.58	NaN	23.58	23.57
A0A452F5S1	SVIP	Small VC	23.48	23.17	23.86	23.67	24.11	24.11	23.21	23.18
A0A452EWJ5	EIF3A	Eukaryot	25.65	24.8	23.87	NaN	NaN	NaN	NaN	NaN
A0A452E7J6	CD14	Monocyte	26.35	26.62	23.87	23.86	25.88	26.05	27.73	27.51
A0A452FNY5	SAR1B	Secretic	28.34	28.45	23.9	24	25.11	25.38	27.34	27.31
A0A452E9C0	RPL17-C	RPL17-C1	28.13	28.08	23.91	24.09	NaN	NaN	22.31	22.69
A0A452EH63	ALDOA	Fructose	28.39	28.24	23.92	24.03	21.43	23.81	26.86	27.11
A0A452EK19	YBX1	CSD_1 do	23.85	23.98	23.93	24.13	NaN	NaN	NaN	NaN
A0A452F9F6	SERPING	Serpin f	NaN	NaN	23.94	23.93	25.76	25.02	NaN	22.61
A0A452G0Y0	PTP4A2	Protein	27.07	26.7	23.94	24.27	24.71	25.04	26.26	26.16
A0A452FVT4	APOC4	Uncharac	23.88	24.09	23.95	24.04	23.2	22.77	24.6	24.04
A0A452GAX8	NCSTN	Nicastri	27.4	26.73	23.95	24.68	24.59	23.23	26.11	27.08
A0A452G3B2	SEC61B	Protein	25.96	26.99	23.96	24.04	NaN	23.79	25.88	25.37
A0A452EB98	LOC1086	SERPIN c	NaN	NaN	23.97	23.96	NaN	NaN	NaN	NaN
A0A452DME8	SFN	Stratifi	26.4	26.33	23.97	23.97	23.2	23.24	25.82	25.98
A0A452DMN7	LOC1086	40S ribo	27.32	27.69	23.98	23.84	20.22	20.76	NaN	21.42
A0A452DUH2	ADAM10	ADAM met	26.49	26.85	23.99	24.6	24.64	24.85	25.3	25.3
A0A452FF94	GLRX	Glutared	26.96	26.79	24	24.21	22.78	NaN	25.57	25.55
A0A452EUZ1	GPI	Glucose-	24.05	23.85	24.01	24.14	22.95	23.08	24.43	24.77
A0A452FH78	AHSG	Alpha 2-	23.06	22.26	24.02	24.21	22.5	22.85	24.99	24.71
A0A452EEG7	LOC1021	Uncharac	26.14	25.99	24.02	23.83	NaN	NaN	22.78	22.89
A0A452E9L8	N/A	Ig-like	23.43	26.85	24.02	23.64	25.33	25.29	24.99	NaN
A0A452G3T1	LMAN1	Lectin,	28.96	28.84	24.03	23.9	NaN	NaN	26.4	26.06
A0A452FPC2	ACSL1	Acyl-CoA	29.5	29.15	24.04	24.16	26.87	26.81	28.33	28.37
A0A452F0H2	ALYREF	Aly/REF	NaN	22.66	24.04	24.11	NaN	NaN	NaN	NaN
A0A452DMW4	RPL13	60S ribo	27.29	27.63	24.05	24.32	NaN	NaN	22.01	22.39
A0A452FZ28	TXNDC5	Thioredo	27.13	27.67	24.05	24.36	NaN	NaN	24.95	25.47
A0A452DVY2	PLLP	Plasmoli	27.93	28	24.06	23.71	23.49	23.6	24.26	26.45
A0A452EAS1	GNA11	G protei	28.01	27.92	24.09	22.63	24.98	25.24	27.55	26.79
A0A452EMZ1	RPL36	60S ribo	25.58	25.67	24.1	23.43	NaN	NaN	23.21	23.06
A0A452FHN5	TPD52L2	TPD52 li	25.96	26.1	24.12	24.3	24.12	23.92	25.84	25.49
A0A452G9C5	STUM	Stum, me	25.55	25.39	24.12	24.06	26.12	26.12	26.74	26.92
A0A452F257	MGP	Matrix (	NaN	NaN	24.13	24.05	20.48	20.78	NaN	NaN
A0A452E8Q8	LOC1021	Ribosoma	26.48	26.63	24.13	24.19	NaN	NaN	NaN	23.25
A0A452DYG0	TMED9	GOLD dom	27.35	27.1	24.14	24.13	22.43	22.17	23.69	24.57
A0A452E481	CNTFR	Ciliary	26.03	26.1	24.15	23.89	25.74	26.32	26.99	27.04
A0A452G9M1	ENG	Endoglin	27.88	27.16	24.15	23.85	25	25.37	26.81	26.93
A0A452DTY2	SLC25A5	Uncharac	27.88	27.36	24.17	24.67	NaN	NaN	23.87	22.92

A0A452E2Q7	JAM3	Junctior	24.7	24.53	24.18	24.52	23.76	23.65	24.48	24.08
Q9TV01	PrP	Major pı	25.54	25.53	24.2	24.69	26.07	26.17	25.35	25.52
A0A452F9L1	RAB8B	RAB8B, n	26.96	26.91	24.23	24.71	24.87	24.8	26.35	26.21
A0A452G9E6	FOLR3	Folate_r	27.13	27.2	24.23	24.05	25.23	25.4	29.27	29
A0A452ET55	TPI1	Triosepł	23.41	24.82	24.24	23.37	21.55	21.8	25.98	25.44
A5HEW3	CYB5	Cytochrc	27.28	27.54	24.26	23.99	20.99	NaN	25.62	24.89
A0A452EJE8	GNA13	G protei	28.79	28.54	24.26	23.11	26.83	26.53	27.89	27.64
A0A452FX42	RPS9	Ribosoma	28.33	28.32	24.27	24.57	NaN	NaN	23.18	21.99
A0A452EWC8	SLC16A1	MFS doma	28.33	27.99	24.29	24.5	24.47	24.93	26.88	26.94
A0A452EJ02	NPM1	Uncharac	NaN	NaN	24.31	24.75	NaN	NaN	NaN	NaN
A0A452E047	LOC1086	Ig-like	27.09	26.87	24.31	24.37	22.98	22.85	25.38	25.43
A0A0U5A2G6	N/A	Kappa ca	NaN	NaN	24.34	24.28	23.04	23.45	NaN	22.55
Q9XSL1	csn1s2	Alpha s2	23.42	23.75	24.34	24.61	23.35	23.16	25	25.01
A0A452FLW9	CD109	CD109 mc	28.46	28.12	24.35	24.74	25.63	25.49	29.67	29.56
A0A452E972	RPL24	Ribosoma	25.3	25.3	24.35	24.21	NaN	NaN	NaN	21.5
A0A452ERN5	CD82	Tetraspa	25.21	25.15	24.35	24.39	24.36	24.33	24.03	23.39
A0A2U8URJ6	N/A	Beta-cae	NaN	22.06	24.35	24.48	25.12	24.23	26.48	25.57
A0A452F069	RPL18	Ribosoma	27.96	27.9	24.35	25.09	NaN	21.87	24.19	23.56
A0A452FHV8	CAP1	Adenylyl	25.34	25.7	24.37	23.95	21.71	21.52	24.73	24.73
A0A452F0A8	N/A	40S ribc	24.57	24.51	24.37	24.55	NaN	NaN	NaN	NaN
A0A452FXX0	MDH2	Malate c	25.03	25.21	24.38	24.66	NaN	NaN	24.09	23.48
A0A452DTL7	SSR4	Signal s	29.45	29.35	24.39	24.34	21.82	21.6	26.89	26.57
A0A452F4R6	IDH1	Isocitra	29.6	29.77	24.4	24.37	25	23.7	26.85	27.18
A0A452EM38	TMED2	GOLD dom	27.32	25.96	24.4	24.49	NaN	NaN	24.91	24.85
A0A452FR97	PGAM1	Phosphog	24.69	25.13	24.4	24.41	22.32	23.07	25.88	25.77
A0A452E037	MARCKS	Myristoy	26.73	26.41	24.43	24.38	23.96	24.75	26.15	26.17
A0A452F4D3	LOC1021	Cystatir	26.72	26.83	24.46	24.19	NaN	NaN	25.36	24.7
A0A452F1F7	RPLP2	Ribosoma	29.45	29.2	24.48	24.66	20.47	21.23	30.36	30.27
A0A452E7Q3	TMPO	Thymopoi	NaN	NaN	24.49	24.56	NaN	NaN	NaN	NaN
A0A452G724	GDI2	Rab GDP	29.55	29.81	24.5	24.89	26.4	26.28	27.86	27.92
A0A452F4D1	LOC1021	Lipocln_	NaN	NaN	24.51	24.67	26.57	26.47	22.46	22.36
A0A452F333	CDH1	Cadherir	24.22	23.68	24.51	24.81	23.78	24.13	24.42	24.14
A0A452EX01	ALDH3B2	Aldehyde	27.83	27.64	24.51	24.23	26.26	26.25	27.2	27.01
A0A452F195	SDCBP	Syndecar	23.61	23.66	24.52	24.54	24.01	24.15	25.14	25.15
A0A452DXA6	STEAP4	STEAP4 n	29.06	28.98	24.53	24.62	26.2	26.62	27.56	27.79
A0A452DUC7	LOC1021	Uncharac	25.08	25.22	24.53	24.71	23.07	23.49	25.33	25.18
A0A452EAY9	N/A	Uncharac	26.32	26.24	24.54	24.87	NaN	NaN	22.72	24.3
A0A452G867	KRAS	KRAS prc	28.38	28.34	24.55	24.96	26.05	25.98	27.32	26.93
A0A452FAW4	LBP	Lipopoly	26.12	26.35	24.55	24.8	25.76	25.6	27.25	27.16
A0A452DYZ1	LOC1086	Peptidyl	25.71	26.02	24.56	24.93	24.28	24.02	24.73	24.87
A0A452FXC9	F2	Prothron	24.21	24.22	24.57	24.89	25.01	25.03	27.01	27.01
A0A452DQM6	HSPD1	Heat shc	26.68	26.56	24.57	24.74	NaN	NaN	25.83	25.8
A0A452F7J5	MS4A3	Uncharac	NaN	NaN	24.58	24.91	NaN	NaN	NaN	22.13
A0A452FTM7	SH3BGRL	SH3 doma	25.22	24.68	24.58	24.58	22.75	NaN	24.96	24.97
A0A452EU77	PKM	Pyruvate	24.99	24.69	24.6	24.63	24.24	24.04	24.69	25.04
A0A452EZ94	RPS20	Ribosoma	27.77	27.69	24.6	24.72	23.18	23.12	24.7	24.6
A0A452FEZ7	LOC1021	Uncharac	29.73	29.82	24.61	24.62	26.8	26.75	28.51	28.73
A0A452FJF9	TF	Transfer	NaN	NaN	24.63	24.6	25.02	25.23	NaN	NaN
A0A452G9J7	RPS7	40S ribc	28.14	28.41	24.63	23.55	21.1	NaN	NaN	NaN
A0A452FTY4	LOC1021	Serum an	24.82	24.78	24.66	24.36	24.94	24.81	24.97	25.01

A0A452EI94	MAP1B	Microtuk	24.11	24.32	24.69	NaN	NaN	24.23	NaN	24.39
A0A452EME0	KRT14	Keratin	22.4	22.35	24.71	24.14	22.01	NaN	NaN	NaN
A0A452EUX2	PRDX6	Thioredc	26.95	26.78	24.72	25.29	24.28	24.52	25.3	24.9
A0A452ENP8	DNAJB11	J domair	24.23	24.4	24.73	24.87	23	23.22	23.12	23.59
A0A452DK35	SRGN	Serglyci	25.53	25.3	24.75	24.75	21.93	NaN	25.96	26.15
A0A452DM15	CANX	Calnexir	29.37	29.35	24.76	24.74	21.46	21.39	25.52	26.53
A0A452F1S9	N/A	40S ribc	24.77	25.24	24.77	24.69	NaN	NaN	22.96	23.1
A0A452DTI8	ATP6AP1	ATPase I	22.91	23.08	24.79	24.62	21.98	NaN	24.81	25.82
A0A452ESB8	ACTR3	Uncharac	25.76	24.98	24.81	24.8	22.82	22.42	24.17	24.53
A0A452F7F0	SPCS3	Signal p	26	25.56	24.84	24.13	23.84	23.95	24.81	25.41
A0A452G1I8	RPL26	Ribosoma	27.98	28.17	24.86	24.73	21.54	21.08	21.66	21.09
A0A452DT47	MYL6B	Myosin l	25.84	25.37	24.86	24.68	20.71	20.99	25.46	25.51
A0A452F5L1	PRSS8	Serine p	25.63	25.09	24.88	25.11	25.82	25.63	25.36	25.11
A0A452EYJ1	PEBP1	Uncharac	27.35	27.26	24.9	24.61	24.74	23.35	26.05	26.47
A0A452G9U0	RAB35	RAB35, m	29.33	28.81	24.92	25.78	27.3	27.37	27.86	27.91
A0A452FA73	RPL27	60S ribc	27.73	27.87	24.96	25.3	NaN	NaN	24.47	24.5
Q5VI41	ITGB2	Integrir	26.28	26.18	24.96	25.13	23.19	23.45	25.81	25.93
I1W1N1	RAP1B	Ras-rela	27.64	27.59	24.98	24.46	25.57	25.61	27.24	27.04
A0A452FNM9	TMED10	Transmen	27.86	27.45	25	24.74	NaN	NaN	24.23	23.93
A9LM14	RAB11B	RAB11B	26.72	26.44	25.01	24.95	25.56	25.39	25.12	25.38
A0A452FL64	RETN	Resistir	27.52	27.33	25.03	25.09	21.41	21.15	21.35	21.5
A0A452EZ30	LRG1	Leucine	21.52	22.11	25.04	25.38	25.45	25.94	28.71	28.54
A0A452F117	N/A	Ig-like	26.46	27.67	25.04	25.3	26.33	26.43	25.84	25.74
A0A452EHN7	LMNB1	Lamin B1	24.81	24.82	25.05	25.29	NaN	NaN	NaN	NaN
A0A452FPT0	MARCKSL	MARCKS l	27.71	27.6	25.07	25.01	25.28	25.42	27.26	27.07
A0A452G5D6	PLSCR1	Phosphol	26.17	26.06	25.07	24.85	27.4	27.8	26.4	26.73
A0A452DTR7	BGN	Biglycar	29.59	29.18	25.11	26.13	26.61	26.57	30.46	30.34
A0A452F6P6	KRT8	Keratin	26.53	26.65	25.12	25.3	24.87	22.91	22.91	23.82
A0A452F7S2	RAB25	RAB25, m	29.05	29.02	25.12	25.04	24.85	24.88	26.48	26.15
A0A452E480	LDHB	L-lactat	24.67	25.1	25.12	24.7	22.63	22.13	25.62	25.49
A0A452FBH0	BSG	Uncharac	28.34	28.22	25.13	25.29	25.35	25.25	27.44	27.09
A0A452ETA1	YWHAB	Tyrosine	28.14	28.03	25.16	25.06	23.83	24.04	27.39	27.66
A0A452ETK3	TPD52	Uncharac	26.25	26.35	25.18	25.27	24.63	24.54	26.15	25.96
A0A452FS72	YWHAE	14_3_3 c	28.52	28.19	25.2	24.86	24.33	24.49	27.8	27.58
A0A452DVP7	LCP1	Lymphocy	22.8	22.74	25.2	25.73	23.37	23.71	25.24	23.74
A0A452FD52	PDIA4	Protein	28.3	28.39	25.21	25.78	21.7	21.4	27.47	27.56
A0A452FYY3	APOA2	Apolipop	25.25	27.19	25.23	25.31	25.05	25.07	28.52	27.24
A0A452G5R6	LOC1021	Uncharac	27.05	27.21	25.25	25.31	24.86	24.79	27.89	27.95
A0A452E3D3	PLIN4	Perilipi	NaN	24.23	25.28	25.46	24.83	24.69	26.05	26.08
A0A452E1U8	GNAI2	G protei	28.99	28.91	25.29	24.96	26.9	26.85	27.92	27.95
A0A452EIH8	NUCB1	Nucleobi	19.53	19.71	25.31	25.36	20.65	21.22	26.57	26.47
A0A452FJM9	ACSL3	Acyl-CoA	29.22	28.89	25.33	25.37	27.07	27.01	28.41	28.17
A0A452EQ33	CHMP4B	Charged	26.57	27.18	25.34	25.5	NaN	23.52	25.94	25.75
A0A452FFI1	EHD1	Uncharac	29.1	28.66	25.37	24.99	25.32	25.46	26.99	27.18
A0A452EK18	ITGAM	Integrir	25.28	24.97	25.37	25.58	22.23	NaN	25.16	25.4
A0A452E8V5	EEF1D	Uncharac	28.19	28.12	25.37	25.4	NaN	NaN	26.01	25.71
A0A452EA05	AGR2	Anterior	26.82	26.23	25.38	26.06	25.18	25.73	26.15	26
A0A452ETY3	GNG5	Guanine	27.96	28.21	25.45	25.47	26.26	26.68	26.53	26.45
A0A452E455	LOC1021	Uncharac	26.26	26.22	25.46	25.97	28.64	28.63	27.97	27.74
A0A452G2C1	TMBIM1	Transmen	26.84	26.92	25.46	25.5	24.49	24.75	26.11	25.96

A0A452E7B9	ACTN4	Actinin	26.33	26.06	25.48	24.48	20.94	21.79	23.23	23.29
A0A452F0B7	MPO	Uncharac	25.16	24.88	25.49	25.75	21.74	NaN	25.11	24.61
A0A452E6P7	CHMP2A	Charged	27.14	27.34	25.5	25.6	23.34	23.67	26.06	25.72
A0A452EWE8	CAPNS1	Calpain	27.34	27.02	25.51	25.69	22.9	21.75	27.05	27.15
A0A452EP46	RPL6	60S ribo	28.34	29.2	25.51	24.57	21.99	NaN	24.86	24.92
A0A452EF34	VAMP3	Vesicle	25.65	25.81	25.51	25.77	24.31	24.51	25.2	25.43
A0A452E0L3	LOC1021	Ribosoma	28.48	28.46	25.54	26.37	20.28	21.28	20.37	NaN
A0A452EGE3	LOC1021	Uncharac	NaN	NaN	25.54	25.54	27.3	27.26	NaN	NaN
A0A452ERK1	GNG12	Guanine	29.38	29.6	25.55	26.06	27.81	27.62	27.81	27.98
A0A452FNC0	SCCPDH	Saccharo	29.12	29.26	25.55	25.81	27.37	27.37	28.02	28.12
A0A452DLZ9	HEBP1	Heme bin	28.43	28.4	25.56	25.61	25.91	26.03	28.05	28.12
A0A452FSN2	UXS1	Uncharac	27.02	27.1	25.59	25.48	25.24	25.66	27.46	27.17
A0A452DS03	LOC1086	Uncharac	29.59	29.57	25.64	25.99	27.81	27.86	28.6	28.56
A0A452FSE1	RPS13	Ribosoma	28.46	28.52	25.66	25.68	21.35	21.93	24.07	23.88
A0A452FJ33	CD44	CD44 mol	21.82	22.57	25.67	25.93	NaN	21.21	23.6	NaN
A0A452E104	PGK1	Phosphog	28.21	27.68	25.68	25.79	23.49	24.95	28.7	28.81
A0A452EMK0	EFHD2	EF-hand	NaN	NaN	25.68	25.74	NaN	NaN	NaN	NaN
A0A452ENM8	WRN	WRN RecQ	23.96	21.48	25.7	25.65	26.37	26.26	25.94	25.89
A0A452DXW0	DHRS1	Dehydrog	30.03	29.9	25.71	25.92	27.39	27.23	28.31	28.4
A0A452DPW0	CHI3L1	Chitinas	27.15	27.1	25.72	25.54	26.78	26.76	28.07	27.99
A0A452EBJ3	RPS3	Ribosoma	30	30.02	25.73	25.25	24.64	23.92	25.92	26.22
A0A452FSZ2	DNAJC3	DnaJ hea	28.11	28.05	25.74	25.9	24.75	24.42	27.54	27.45
A0A452DW39	N/A	60S ribo	23.68	24.8	25.8	25.84	NaN	NaN	NaN	NaN
A0A452FNJ2	HNRNPA2	Heteroge	23.28	23.12	25.82	25.88	NaN	20.84	22.1	22.37
A0A452FVD3	RAC1	Rac fami	28.16	28.23	25.84	26.25	27.17	27.1	27.91	27.98
A0A452FKN8	TKT	Transket	29.83	29.56	25.85	25.49	26.13	26.27	28.6	28.49
A0A452G2R8	RAB5A	Uncharac	28.33	28.26	25.87	25.78	26.91	26.99	26.61	26.79
A5JST4	RPS10	Ribosoma	27.61	27.66	25.87	25.84	21.62	NaN	22.55	NaN
E9NRZ3	B4GALT1	Beta-1,4	26.59	26.95	25.88	25.94	26.92	26.89	27.54	27.47
A0A452FSD4	RPS15	Uncharac	27.26	27.18	25.9	25.49	NaN	NaN	NaN	NaN
A0A452EXQ4	STX3	Syntaxin	28.79	28.77	25.94	25.94	26.47	26.42	27.68	27.56
A0A452EN82	ATP5F1A	ATP synt	28.81	29.69	25.96	26.16	22.39	21.63	26.56	26.34
A0A452F116	HYOU1	Hypoxia	28.06	28.27	25.96	25.9	23.15	24	26.7	26.63
A0A452FJW1	SSR1	Signal s	28.35	28.04	25.98	25.7	NaN	21.49	24.74	24.13
A0A452FT08	N/A	Uncharac	26.04	26.8	25.99	25.94	21.91	21.86	23.26	23.4
A0A452FKL3	SDS	Serine d	27.67	27.78	26	25.92	24.65	24.75	27.87	28.08
A0A452DY90	CD151	Tetraspa	27.24	27.25	26.01	26.19	26.4	26.39	27.62	27.43
A0A452FMZ7	HSP90AA	Heat sho	27.84	27.94	26.01	25.72	21.27	22.65	26.36	26.15
A0A452F0K7	TPM3	Tropomyo	26.62	26.57	26.02	25.88	NaN	NaN	25.32	25.4
A0A452FAH1	CKAP4	Cytoskel	27.9	27.77	26.03	25.83	NaN	NaN	24.18	23.83
A0A452EHC3	RALA	RAS like	28.77	28.82	26.03	26.11	26.61	26.59	28.82	28.58
A0A452G1I9	EHD4	EH domai	29.6	29.57	26.03	26.1	27.68	27.72	28.71	28.58
A0A452ER34	RAB1B	RAB1B, m	28.55	28.06	26.03	25.74	26.61	26.57	27.4	27.16
Q6T5B8	MSTN	Growth/d	25.25	25.62	26.08	26.13	27.09	26.96	25.41	25.33
D6PX62	Crisp3	Cysteine	27.17	26.84	26.09	26.45	27.84	27.99	27.42	27.69
A0A452DSV0	HSD17B7	Hydroxys	29.72	29.67	26.1	26.03	28.04	28.16	28.71	28.63
A0A452DQP6	ACTC1	Actin al	29.39	29.6	26.11	26.15	25.09	25.67	27.26	27.37
A0A452F327	HSP90AB	Heat sho	29.52	29.56	26.12	26.36	NaN	23.31	26.75	26.43
A0A452G3C9	CALU	Calumeni	27.13	27.13	26.12	26.13	NaN	NaN	26.4	26.38
A0A452DUB7	PTHLH	Uncharac	26.15	26.52	26.14	26.07	26.24	26.83	25.67	25.02

Q0GC71	TLR2	Toll-lik	29.68	29.73	26.14	26.32	27.23	27.3	28.7	28.76
A0A452EPC6	N/A	Uncharac	29.66	29.78	26.17	26.34	27	26.81	29	29.06
A0A452DVG8	PDIA6	Protein	28.08	28.15	26.17	26.32	24.31	24.58	27.56	27.52
A0A452F3Z8	TPD52L1	TPD52 li	28.07	27.69	26.17	25.97	25.34	25.56	26.83	27.12
A0A452ERR1	TMEM263	Transmem	28.08	27.88	26.17	26.16	26.72	26.69	29.15	29.07
A0A452G7G8	JCHAIN	Joining	26.86	26.88	26.21	26.21	26.23	26.65	27.78	27.74
A0A452DYD4	LOC1021	Uncharac	29.17	29.5	26.24	26.35	27.24	27.1	28.71	27.65
A0A452E7H3	LOC1021	H15 doma	24.9	24.74	26.27	25.53	NaN	23.2	NaN	24.21
A0A097C2Z1	YWHAZ	Tyrosine	28.93	28.99	26.28	25.88	24.61	24.41	28.18	28.1
A0A452F854	LOC1021	SERPIN c	22.27	NaN	26.28	26.04	25.27	26.16	23.62	22.73
P18626	CSN1S1	Alpha-S1	NaN	22.27	26.32	26.69	25.76	25.75	25.72	25.49
A0A452EHI7	N/A	Uncharac	28.86	28.29	26.33	25.53	27.1	27.52	28.42	28.24
A0A452G0B8	OS9	OS9 endo	28.05	28.03	26.33	26.25	23.35	23.3	27.89	28.03
A0A452FHI3	REEP5	Receptor	26.17	26.47	26.36	26.46	21.49	21.38	24.04	24.58
A0A452ESU7	HSPB1	Heat sho	26.3	26.28	26.38	26.55	25.47	25.57	26.39	26.41
A0A452DSB1	ANXA2	Annexin	29.16	29.16	26.41	26.43	27.1	27.1	29.01	28.93
A0A452E1B3	SIL1	SIL1 nuc	25.51	25.66	26.43	26.43	26.21	26.45	27.24	27.47
Q06B57	FASN	Fatty ac	30.34	30.22	26.43	26.39	27.55	27.66	29.17	29.09
A0A452E3K5	HIST2H2	Histone	NaN	NaN	26.44	28.19	NaN	23.39	NaN	NaN
A0A452E3Q7	RAB5B	RAB5B, m	28.36	28.63	26.45	26.61	27.59	27.37	28.04	27.5
A0A452EU55	GNG5	Guanine	26.12	25.95	26.46	26.7	24.92	25.12	26.67	26.47
A0A452F1W3	THBS1	Thrombos	26.14	26.15	26.46	26.41	25.76	25.89	27.3	27.38
A0A452E0H4	MYL12B	Myosin l	26.49	26.02	26.47	26.31	NaN	23.14	26.02	25.95
A0A452DS67	HIST2H2	Histone	NaN	NaN	26.49	25.64	NaN	NaN	NaN	NaN
A0A452FHR6	SLC6A14	Transpor	28.93	28.9	26.51	26.05	26.75	26.47	28.71	28.33
A0A452E3I1	LOC1065	Histone	NaN	NaN	26.53	26.54	NaN	NaN	NaN	NaN
A9LM13	rab14	RAB14	29.27	28.97	26.53	26.55	27.46	27.3	27.92	27.96
A0A452FP90	GANAB	Glucosid	27.97	27.83	26.54	26.71	26.64	26.68	28.22	27.9
A0A452FGJ8	DPP4	Dipeptid	27.25	27.38	26.54	26.66	27.19	27.23	28.22	28.35
A0A452F730	SAR1A	Secretio	29.73	29.71	26.54	26.19	27.6	27.75	28.95	28.72
A0A452E5I0	HIST1H1	Histone	22.72	23.49	26.54	26.8	21.91	NaN	23.42	23.65
A0A452G9Y2	ANXA4	Annexin	27.37	27.32	26.58	26.27	26.71	26.8	27.78	27.59
A0A452E7B0	LOC1021	Arp2/3 c	26.53	26.37	26.6	24.9	23.02	23.03	23.68	24.41
A0A452G587	PFN1	Profilin	27.02	26.66	26.63	26.48	25.43	25.44	28.47	28.65
A0A452F2C6	EZR	Ezrin	28.33	28.43	26.63	26.93	25.93	25.98	27.38	27.52
A0A452FHJ3	GSN	Gelsolin	30.52	30.53	26.63	26.66	27.25	27.37	29.34	29.36
A0A452EC18	HIST1H1	Histone	NaN	21.21	26.63	25.83	NaN	NaN	NaN	NaN
A0A452G899	DNAJC5	J domain	27.41	27.43	26.65	26.43	27.32	27.28	27.93	27.89
A0A452DZE7	S100A11	Protein	26.15	26.67	26.66	26.18	NaN	23.97	26.48	25.59
A0A452E4U4	NT5E	5'-nucle	29.81	29.72	26.7	26.54	28.09	28.15	29.66	29.41
A0A452FVN5	RPL31	Uncharac	28.46	28.05	26.7	26.62	NaN	NaN	23.9	23.93
A0A452ECE5	ERP29	Endoplas	28.85	28.6	26.73	26.72	22.76	22.71	26.56	26.71
A0A452ELY6	NSDHL	NAD(P) d	31.9	31.83	26.73	28.34	29.36	29.2	30.65	30.56
A0A452F3L3	AGPAT1	1-acyl-s	23.85	NaN	26.74	26.89	25.8	25.59	25.96	25.84
A0A452F449	LOC1021	Uncharac	24.86	24.85	26.74	26.74	25.72	25.66	29.2	29.22
A0A452EQX6	DBI	Diazepam	27.95	27.53	26.78	26.93	23.51	25.05	26.58	26.47
A0A452FJ34	H2AFY	Core his	19.98	21.36	26.81	27.6	NaN	NaN	20.04	19.81
A0A452FGW6	N/A	E2F_TDP	29.61	29.7	26.82	26.86	24.04	26.68	28.97	28.93
A0A452FYV6	CFL1	Cofilin	28.73	28.6	26.83	26.65	25.89	25.79	27.6	27.49
I1VE56	RAB8A	RAS onco	29.89	29.89	26.89	26.84	27.85	27.65	28.22	28.26

A0A452FPP9	CD47	CD47 mol	28.07	28.14	26.9	26.88	26.45	26.57	28.45	28.24
A0A452E2H7	EEF1A1	Elongati	30.97	30.89	26.9	26.82	27.61	27.49	29.02	28.86
A0A452EHS7	SDF4	45 kDa c	NaN	NaN	26.9	27.17	19.59	19.46	19.59	19.82
A0A452EWT8	NLRP12	NLR fami	29.88	29.41	26.93	26.12	25.66	25.55	28.12	26.94
A0A452FQC0	LOC1021	Uncharac	27.36	27.4	26.99	27.29	25.33	25.31	28.39	28.44
A0A452DVM2	RAB10	RAB10, m	29.11	28.87	26.99	26.94	27.1	27.31	28.07	28.1
A0A452F540	HMGB1	Uncharac	24.95	24.92	27	27.14	22.51	20.79	24.92	24.63
A0A452EEF1	RAB5C	Uncharac	30.14	30.16	27	26.79	28.19	28	29.04	28.77
A0A452F4L3	FGA	Fibrinog	28	28.1	27.02	27.2	29.23	29.14	27.31	27.29
A0A452FN53	CHP1	Calcineu	31.33	31.25	27.05	27	26.26	26.61	31.3	31.43
A0A452E2P1	N/A	Uncharac	27.39	27.49	27.08	26.9	27.78	27.81	28.97	28.96
A0A452EXL2	VAMP8	Vesicle	27.88	27.81	27.08	27.07	25.82	25.98	26.93	26.95
A0A452G271	RPS18	40S ribo	29.14	28.92	27.09	26.75	23.69	23.95	25.83	26.32
A0A452DZ35	AZU1	Azurocic	26.55	NaN	27.09	27.14	24.95	25.11	NaN	24.55
A0A452G9Q1	YKT6	Uncharac	29.73	29.39	27.09	27.21	27.46	27.73	28.6	28.48
A0A452DZU0	LOC1021	SERPIN c	NaN	NaN	27.1	27.28	27.04	25.89	22.23	21.03
A0A452E8X3	ARHGDIB	Rho GDP	21.11	NaN	27.11	26.91	22.92	22.19	23.07	22.87
A0A452F4J2	SDC4	Syndecar	26.6	26.66	27.13	26.99	25.59	25.8	26.82	26.83
A0A452FKT0	ANXA5	Annexin	28.78	28.86	27.16	27.24	26.79	26.91	29.2	29.16
A0A452ED08	LRPAP1	LDL rece	26.78	27.62	27.18	27.01	NaN	21.29	24.5	24.73
A0A452E8W7	ANXA3	Annexin	28.16	27.96	27.19	27.14	25.85	25.83	28.72	28.8
A0A452FXR8	VNN2	Vanin 2	27.06	27.01	27.23	27.54	28.52	28.65	28.64	28.3
A0A452F8I7	RALB	RAS like	30.09	29.98	27.28	27.35	27.19	27.23	28.58	28.62
A0A452EZ43	BPIFB1	BPI folc	29.64	29.78	27.29	27.53	30.08	29.83	30.78	30.6
A0A452G8E9	N/A	Uncharac	NaN	26.32	27.29	26.27	23.81	23.87	25.99	25.25
A0A452DX04	N/A	Peptidyl	30.3	30.09	27.31	27.01	27.79	27.56	29.93	29.9
A0A452E734	CALM2	Calmodul	28.76	28.9	27.32	27.08	25.28	25.83	29.54	29.29
A0A452DYR7	CST3	Cystatir	25.47	25.56	27.32	26.69	27.36	27.28	27.08	27.17
A0A452ED29	PIGR	Polymeri	27.44	27.34	27.36	27.65	27.7	27.85	28.74	28.76
A0A452F1G6	RAP1A	RAP1A, m	30.54	30.5	27.41	27.27	28.26	28.34	29.8	29.69
A0A452ENB6	C9	Compleme	26.59	26.65	27.42	27.35	28.92	29.03	29.26	29.25
A0A452G286	N/A	Uncharac	28.94	28.77	27.46	27.57	24.76	24.77	25.72	25.68
A0A452EGM8	VAT1	Vesicle	30.76	30.7	27.48	27.5	28.57	28.7	30.13	29.97
A0A452FN24	KRT3	Keratin	20.94	NaN	27.48	27.32	23.94	24.47	23.26	23.67
A0A452E0H5	CYB5R3	NADH-cyt	31.16	31.08	27.48	27.45	28.7	28.71	30.6	30.58
P85170	N/A	Catheli	26.21	26.35	27.49	26.72	27.26	27	26.81	26.89
A0A452F8V5	ARF3	ADP ribc	31.09	30.77	27.51	27.9	29.51	29.4	29.18	29.23
P0CH25	HBA1	Hemoglob	27.05	27.06	27.52	27.51	27.02	27.25	27.41	27.26
A0A452EE69	N/A	Ig-like	27.77	27.59	27.52	27.48	28.27	28.37	29.61	29.42
A0A452FCF9	SFT2D2	Vesicle	28	27.95	27.52	27.1	24.8	25.46	27.32	27.33
A0A452E0U0	CD81	Tetraspa	27.84	27.63	27.54	27.85	26.98	26.9	28.62	28.5
A0A452E1G9	HSP90B1	Heat sho	30.84	30.84	27.59	27.79	25.17	25.27	28.97	28.3
A0A452EZ57	GGT1	Uncharac	28.25	28.28	27.61	27.37	28.13	28.14	29.15	28.8
A0A452G885	KRT4	Keratin	24.68	24.55	27.64	26.92	24.78	24.88	NaN	NaN
A0A452FPK7	LOC1021	Serum am	27.19	26.94	27.67	27.86	27.19	27.19	27.65	27.54
A0A452EM11	APOC3	Apolipop	26.7	26.99	27.67	27.74	27.84	27.53	27.9	27.75
A0A452ET78	LOC1021	SERPIN c	NaN	NaN	27.69	27.63	24.77	24.67	21.66	NaN
A0A452E6V0	HMGB2	Uncharac	27.35	27.24	27.73	27.82	24.57	23.77	26.39	26.37
A0A452E573	ATP5F1B	ATP synt	30.34	30.37	27.76	27.69	NaN	21.74	28.56	29.67
A0A452DMR1	RAB7A	Uncharac	31.14	31.12	27.76	27.75	28.98	28.87	30.36	30.28

A0A452EEM4	PGLYRP1	Peptidog	30.93	30.77	27.8	27.47	27.72	28.25	29.14	29.16
A0A452FTQ5	H2AFV	Histone	NaN	NaN	27.83	27.83	22.83	22.94	23.67	24.1
A0A452E2R6	LOC1021	Uncharac	28.63	28.45	27.83	28.08	28.23	28.52	28.27	28.57
I1W1N0	RAB2A	Ras-rela	31.26	31.2	27.86	27.82	28.58	28.51	30.1	30.18
A0SWR1	CSN3	Kappa ca	25.46	25.16	27.9	27.85	27.02	27.19	28.43	28.51
A0A452FGY2	LOC1086	MHC_I-li	30.3	30.35	27.93	28.13	29.82	30.15	30.73	30.83
A0A452FNW6	GAPDH	Glyceral	28.97	29.01	27.94	28.08	27.24	27.17	29.02	29.08
A0A452FYB3	KRT19	Keratin	27.34	27.21	27.96	27.69	26.6	26.68	26.56	26.52
A0A075VRE8	CRBP1	Cellular	30.99	30.9	27.98	27.99	28.45	28.34	30.59	30.48
A0A452FB52	DNAJB9	DnaJ hea	28.3	28.15	28	27.85	26.21	26.7	26.63	26.25
A0A452E364	PIEZO2	Piezo-ty	30.92	30.95	28	28.13	26.13	26.16	29.49	29.59
A0A452FWI2	RAB6A	RAB6A, m	31	31.07	28.03	28.04	28.6	28.71	29.45	29.42
A0A452FKB0	HIST1H1	Histone	NaN	24.58	28.05	28.05	22.86	22.82	NaN	NaN
A0A452EER2	HIST2H2	Histone	24.41	24.62	28.05	28.25	22.93	22.93	23.9	23.52
A0A452G855	P4HB	Protein	31.2	31.22	28.05	27.99	25.89	25.95	29.82	29.76
A0A452ED36	BCAP31	B cell r	29.19	29.1	28.11	28.01	21.01	NaN	26.28	25.83
A0A452DZD6	N/A	Ig-like	29.97	29.87	28.12	28.22	29.57	29.29	28.9	28.71
A0A452G1R9	NUCB2	PSEN1	26.8	26.57	28.12	28.31	21.38	22.57	28.48	28.42
A0A452G7F5	N/A	SEA doma	29.03	29.09	28.21	28.43	27.8	27.49	28.31	28.01
A0A452FK82	ARHGDIA	Rho GDP	30.42	30.27	28.24	28.06	27.39	27.21	28.9	28.99
A0A452EZT9	XDH	FAD-binc	32.12	32.4	28.25	28.25	31.02	31.38	27.6	27.91
A0A452FVV2	CIDEA	CIDE-N c	33.05	32.75	28.29	28.14	30.18	30.25	31.29	31.2
A0A452F2T3	SLC28A3	Sodium/n	33.02	32.89	28.34	28.45	28.17	27.99	30.8	30.89
A0A452EAJ1	LOC1021	Uncharac	27.04	26.99	28.36	28.56	28.04	28.04	29.21	28.64
A0A452DLS3	TMEM109	Transmem	25.98	27.36	28.36	27.17	24.42	NaN	26.37	25.14
A0A452E9Y6	LPO	Lactoper	29.45	29.44	28.37	28.55	29.8	29.68	31.1	31.11
A0A452FAW1	MYH9	Myosin h	27.38	27.04	28.37	28.38	NaN	NaN	24.81	24.89
A0A452G3V5	LPL	Lipoprot	31.14	30.88	28.37	28.27	28.76	28.87	31	31.32
M4NBE4	HSPA5	Heat shc	31.13	31.14	28.42	28.51	26.78	27.02	29.77	29.69
A0A452DTY6	SLC39A8	Solute c	29.59	30.38	28.47	28.41	27.6	27.53	29.15	28.55
I1W1N3	RAB18	Ras-rela	32.08	32.26	28.48	28.44	30.18	30.03	30.34	30.13
A0A452FTJ0	CALR	Calretic	30.93	30.95	28.55	28.53	23.47	24.8	29.51	29.54
A0A452F9Y8	PPIB	Peptidyl	28.35	28.14	28.55	28.48	27.26	27.35	29.06	28.96
P82018	CATHL2	Catheli c	23.93	NaN	28.65	28.11	28.03	28.5	28.3	28.46
A0A452DY37	B2M	Beta-2-m	27.6	28.02	28.69	28.48	29.02	28.92	29.32	29.16
A0A452F3X9	PROM2	Prominir	30.91	30.93	28.79	28.84	29.74	29.68	30.8	30.7
A0A452FVY9	MUC15	Mucin 15	29.52	29.58	28.82	28.83	29.48	29.25	29.9	29.82
A9LM10	N/A	RAB1A	32.34	32.36	28.95	28.75	29.66	29.71	30.78	30.77
A0A452ED43	APOA4	Apolipop	27.42	27.12	28.98	29.15	28	28.12	30.52	30.5
A0A452FXA9	MELTF	Melanotr	32.74	32.88	28.99	28.98	29.8	29.77	32.56	32.54
A0A452FE94	VIM	Vimentir	28.92	28.91	29.01	29.09	24.64	24.65	27.08	27.11
A0A452G9K5	KRT1	Keratin	26.27	26.52	29.02	28.99	27.48	27.09	26.09	25.86
A0A452EBK8	RAB11A	RAB11A,	31.6	31.68	29.03	29.28	29.66	29.7	30.28	30.13
A0A452E9N0	STOM	Stomatir	34.64	34.67	29.03	29.3	30.44	30.56	32.37	32.28
A0A452FHZ0	LCN2	Lipocali	29.28	29.48	29.06	29.14	27.35	27.41	27.78	27.91
A0A452E9L6	HSD17B1	Hydroxys	30.87	30.03	29.07	29.16	NaN	28.98	27.71	27.29
A0A452DMF8	KRT5	Keratin	23.2	24	29.21	29.12	27.1	26.06	23.38	22.36
A0A452F5G1	SERPINB	Serpin f	25.01	25.19	29.21	29.16	27.52	26.73	27.41	27.04
A0A452ERT5	SLC34A2	Solute c	31.58	31.57	29.29	29.39	29.57	29.67	30.98	30.79
A0A452ES93	SDC2	Syndecar	29.15	29.36	29.4	29.29	28.89	28.93	29.03	29.01

A0A452DXE2	C3	Compleme	32.11	32.17	29.45	29.41	31	30.99	31.85	31.69
Q865G0	N/A	Beta-act	32.48	32.45	29.58	29.7	28.53	28.71	30.97	31.02
A0A452F7K4	ALB	Serum al	25.16	NaN	29.65	30.01	29.89	30.42	25.47	24.52
A0A452DUG3	Synapto	Synaptos	30.67	30.71	29.69	29.56	30.25	30.27	29.77	29.75
A0A452G0P6	KRT10	Keratin	26.5	26.35	29.71	29.8	27.51	27.66	26.8	27.07
A0A452FYQ9	UBA52	Ubiquiti	31.15	31.05	29.75	29.74	28.76	28.77	29.57	29.4
Q09GP3	ABCG2	ATP-binc	33.87	33.8	29.77	29.76	31.15	31.22	32.55	32.46
A0A452F0Q6	N/A	Uncharac	32.4	32.33	29.77	29.87	30.44	30.64	31.77	31.67
P82017	map34-A	MAP34-A	30.42	30.32	29.93	30.09	29.55	29.53	31.71	31.64
A0A452DYP4	CD9	Tetraspa	30.43	30.79	30.18	30.21	29.91	30.33	31	30.89
A0A452FVG0	PLIN3	Perilipi	31.86	31.98	30.28	30.31	31.26	31.26	32.35	32.27
A0A452E2Y0	LOC1021	Clusteri	31.99	31.88	30.32	30.13	29.54	29.26	32.85	32.75
A0A452FL25	APOE	Apolipop	30.11	30.22	30.34	30.16	31.53	31.5	31.44	31.44
Q9XSQ9	bac7.5	Bac7.5 p	28.46	28.57	30.45	30.36	28.24	28.16	29.37	29.09
A0A452DM79	HIST1H1	Histone	25.8	25.55	30.57	30.71	24.33	23.46	26.73	27.03
A0A452FMU8	MUC1	SEA doma	33.46	33.23	30.7	30.64	29.48	29.74	33.03	33.01
Q9XSQ8	map28	MAP28 pr	30.19	30.05	30.75	30.88	30.52	30.47	32.38	32.28
Q29477	LTF	Lactotra	32.55	32.55	30.86	30.83	31.62	31.6	33.31	33.28
A0A452FG03	ANXA1	Annexin	31.72	31.8	31.03	30.92	30	30.15	32.47	32.43
A1YZ34	XHD	Xanthine	37.26	37.34	31.24	31.12	33.52	33.52	35.49	35.42
A9YUB7	OPN	Osteopor	27.01	27.19	31.39	31.66	29.69	29.8	31.73	31.71
A0A452DXL6	CCDC18	Coiled-c	27.1	27.19	31.53	29.93	29.62	29.49	31.02	30.7
Q6S4N9	H-FABP	Fatty ac	33.9	33.92	31.68	31.67	30.89	31	33.1	33.05
M9TKR5	ADFP	Perilipi	34.86	34.84	31.7	31.87	33.13	33.1	34.53	34.35
A0A452G1U2	CD36	Uncharac	32.82	32.89	31.86	31.97	32.21	32.31	33.34	33.28
A0A452G893	S100A8	S100 cal	27.57	27.22	31.88	31.66	28.69	28.96	30.43	30.32
A0A452EXE8	S100A9	EF-hand	26.97	27.18	32.02	32.06	28.41	28.28	29.94	29.6
A0A452FI14	APOA1	Uncharac	31.57	31.42	32.11	32.05	30.28	30.37	33.18	33.05
A0A452EDZ4	S100A12	Protein	28.6	28.34	32.17	32.23	30	29.79	30.38	30.01
A0A452DT02	LOC1021	Histone	29.68	29.67	32.17	31.66	26.94	26.98	28.81	28.57
A0A452DSE1	HIST1H1	Histone	29	28.99	32.59	32.6	27.38	27.39	29.27	29.24
P85297	MFGE8	Lactadhe	33.95	34.04	33.07	32.95	33.2	33.11	34.92	34.44
A0A452E1V4	LOC1021	Histone	29.66	29.54	33.3	32.94	28.45	28.56	29.31	29.04
A0A452ECD3	LOC1008	Serum am	31.67	32.07	33.4	33.35	32.75	32.86	33.69	33.43
A0A452ECD2	H2AFX	Histone	30.07	30.33	33.5	33.39	28.48	28.25	29.68	29.15
P02756	LGB	Beta-lac	30.9	30.77	33.85	34.19	34.67	34.75	33.76	33.62
A3EY52	BTN1A1	Butyroph	36.93	36.97	33.86	33.83	35.19	35.23	36.76	36.69
A5JSR9	N/A	Serum am	33.52	33.57	33.89	33.36	34.48	34.34	32.7	33.28
A0A452DWU1	LOC1065	Histone	32.13	32.06	34.66	34.64	30.48	30.52	31.72	31.56
A0A452EGX6	GLYCAM1	Glycosyl	34.6	34.8	35.3	35.5	35.77	35.82	35.61	35.33
P33049	CSN1S2	Alpha-S2	32.19	32.49	35.72	35.61	34.89	35.03	34.99	34.87
P33048	CSN2	Beta-cas	33.8	34.05	36	35.93	35.62	35.68	36.3	36.42
A0A452E968	CCT3	T-comple	26.15	26.36	NaN	NaN	19.38	NaN	NaN	20.06
A0A452F5N4	MTPN	Myotroph	21.89	21.74	NaN	20.94	19.54	NaN	NaN	NaN
A0A452GAH3	ATP1B3	Sodium/p	23.71	23.81	NaN	NaN	19.9	20.29	21.63	21.26
A0A452EL91	YARS	Tyrosine	26.17	25.83	NaN	NaN	20.24	NaN	24.44	23.91
A0A452FKE5	LOC1021	Uncharac	29.64	29.86	NaN	20.53	20.33	21.21	28.57	28.43
A0A452DK32	F10	Coagulat	26.31	26.26	NaN	NaN	20.47	21.02	24.89	24.75
A0A452EJN6	CRYAB	Crystall	22.66	21.25	NaN	NaN	20.52	NaN	NaN	NaN
A0A452EEL3	MINPP1	Multiple	NaN	20.91	NaN	NaN	20.78	NaN	24.02	23.46

A0A452EPV9	MIEN1	Migratic	23.22	NaN	NaN	NaN	20.83	20.93	NaN	NaN
A0A452G9K3		Solute c	23.67	23.82	NaN	20.19	20.84	21.03	NaN	NaN
A0A452FL49	N/A	Ferritir	NaN	NaN	NaN	NaN	20.87	NaN	23.44	23.25
A0A452ECJ5	ADGRG2	Adhesior	25.95	26.12	NaN	NaN	20.98	21.2	27.25	26.81
A0A452EF60	CAV2	Caveolir	NaN	NaN	NaN	NaN	21.14	22.15	NaN	NaN
A0A452G0J3	TIMP2	TIMP met	22.63	22.16	NaN	NaN	21.35	22	22.99	22.84
A0A452FRI6	PITPNC1	Phosphat	22.2	22.56	NaN	NaN	21.38	21.56	22.18	22.29
A0A452DL29	OSBPL1A	Oxysterc	22.24	NaN	NaN	NaN	21.48	21.32	21.67	NaN
A0A452FXU9	ATP2B1	Calcium-	25.6	25.6	NaN	NaN	21.58	20.55	23.83	23.86
A0A452DPB0	RAB43	RAB43, n	24.08	23.67	NaN	NaN	21.6	NaN	NaN	NaN
A0A452EJ90	LCAT	Lecithir	NaN	NaN	NaN	NaN	21.71	NaN	NaN	22.49
A0A452FYT7	SERINC5	Serine i	NaN	NaN	NaN	NaN	21.72	NaN	21.85	NaN
A0A452E2I9	SEC22B	SEC22 hc	26.43	26.75	NaN	21.79	21.74	NaN	NaN	NaN
A0A452F7F4	ZDHHC20	Palmitoy	23.65	22.65	NaN	NaN	21.77	NaN	22.29	22.04
A0A452EPF5	PRKG2	cGMP-der	NaN	NaN	NaN	NaN	21.79	21.3	NaN	NaN
A0A452DM56	C16H1or	Uncharac	22.24	NaN	NaN	NaN	21.88	NaN	22.53	22.19
A0A452DKZ3	NAPG	NSF atta	25.3	24.88	NaN	20.37	21.95	21.46	23.24	21.92
A0A452F2N0	TMEM53	Transmen	23.35	23.13	NaN	NaN	21.95	NaN	22.54	NaN
A0A452EYP8	GPRC5B	G protei	23.2	23.14	NaN	21.97	21.95	22.34	23.41	23.06
A0A452DK53	YES1	Tyrosine	22.65	22.57	NaN	NaN	21.97	21.88	22.79	22.71
A0A452FXH6	THY1	Thy-1 ce	NaN	22.03	NaN	21.04	22	22.92	NaN	NaN
A0A452FVL9	HPN	Hepsin	25.36	25.17	NaN	21.84	22.08	22.34	24.72	24.74
A0A452ES30	HPRT1	Hypoxant	24.75	24.05	NaN	NaN	22.15	22.14	22.19	22.46
A0A452F982	NPC2	NPC intr	NaN	NaN	NaN	NaN	22.23	22.14	NaN	NaN
A0A452FX35	CTSA	Carboxyp	NaN	NaN	NaN	NaN	22.23	21.71	21.98	NaN
A0A452EN97	ACACA	Acetyl-C	28.13	27.44	NaN	22.69	22.23	21.9	24.52	24.68
A0A452F1Q3	NME1	Nucleosi	24.92	24.5	NaN	NaN	22.24	NaN	23.81	23.13
A0A452F5B5	STXBP6	Syntaxir	24.04	22.94	NaN	NaN	22.26	22.47	23.8	23.46
A0A452EVI1	RAB6B	RAB6B, n	24.65	24.49	NaN	21.44	22.28	22.34	22.73	22.69
A0A452ERL0	DNAJA1	Uncharac	22.84	22.96	NaN	NaN	22.29	22.49	23.05	23.07
A0A452DP21	FAM114A	Family w	25.31	24.52	NaN	NaN	22.29	21.97	24.55	24.58
A0A452FMB2	SNX1	Sorting	23.57	23.02	NaN	NaN	22.3	22.26	22.86	23.77
A0A452EE35	SLC13A2	Solute c	27.21	27.15	NaN	NaN	22.31	22.76	25.11	24.74
A0A452FNV6	FAM114A	Family w	25.07	24.89	NaN	NaN	22.36	23.33	23.4	23.88
A0A452FSV8	TMEM68	Transmen	24.48	24.42	NaN	NaN	22.39	NaN	22.93	NaN
A0A452FJJ6	FN1	Fibronec	26.83	26.74	NaN	NaN	22.42	24.62	29.29	29.76
A0A452EDB4	ATP13A4	Cation-t	25.16	23.92	NaN	NaN	22.43	22.51	23.38	NaN
A0A452EBX4	N/A	WD_REPEA	NaN	24.58	NaN	NaN	22.52	21.23	22.79	NaN
A0A452ESM5	ALG5	ALG5 dol	26.36	25.99	NaN	22.63	22.54	23.36	24.73	25.12
A0A452E512	ELMOD2	ELMO don	24.59	24.51	NaN	NaN	22.55	22.81	24.21	23.95
A0A452DR48	HRAS	HRas prc	25.16	25.18	NaN	NaN	22.57	22.96	23.8	23.74
A0A452FDS3	SPPL2A	Signal p	24.03	24.16	NaN	NaN	22.6	22.51	24.37	24.31
A0A452EKB3	N/A	Uncharac	25.2	24.69	NaN	NaN	22.61	21.88	24.55	24.52
A0A452F8G0	CREG1	Cellular	22.53	22.15	NaN	20.72	22.62	23.17	22.69	22.96
A0A452EE75	EBAG9	Receptor	21.68	21.41	NaN	NaN	22.65	22.88	NaN	22.02
A0A452G2V7	CROCC	Ciliary	NaN	NaN	NaN	NaN	22.69	22.85	NaN	NaN
A0A452E1J1	NCOR1	Nuclear	NaN	NaN	NaN	NaN	22.73	23.04	NaN	NaN
A0A452FE38	ATP6V1A	ATPase H	26.62	28.92	NaN	NaN	22.77	22.88	24.76	NaN
A0A452DVX2	MVP	Major va	27	27.23	NaN	NaN	22.82	NaN	23.36	22.42
A0A452DL20	TSPAN14	Tetraspa	24.26	25.08	NaN	22.8	22.88	22.88	23.32	23.5

A0A452FTE9	GNAI1	G protei	25.41	24.91	NaN	NaN	22.91	23.16	23.8	23.24
A0A452EKD2	CCT4	T-comple	25.38	25.24	NaN	NaN	22.98	22.6	NaN	NaN
A0A452DND2	LOC1021	VWFA dom	22.63	22.73	NaN	20.6	22.99	23.04	23.75	24.3
A0A452E3Y5	CIB1	Calcium	29.33	29.69	NaN	NaN	23.01	25.46	28.89	29.01
A0A452FH09	GNA14	G protei	23.68	23.3	NaN	NaN	23.02	22.35	23.64	22.82
A0A452DUL2	RAB3A	RAB3A, m	25.93	25.91	NaN	NaN	23.13	22.92	24.02	24.63
A0A452DXP9	DHRS3	Dehydrog	23.75	24.1	NaN	NaN	23.2	23.62	24.15	23.81
A0A452FH30	DNASE1L	Deoxyrib	25.73	25.75	NaN	21.7	23.31	23.33	25.92	25.93
A0A452F2N3	ALDH1A1	Aldehyde	27.65	27.93	NaN	22.09	23.32	22.07	26.51	26.09
A0A452E5L3	PTPRJ	Protein	26.67	26.77	NaN	20.02	23.37	23.29	26.24	26.33
A0A452DZM5	PURA	Purine r	22.85	23.99	NaN	NaN	23.68	NaN	NaN	NaN
A0A452EER1	LDAH	Lipid dr	26.75	26.62	NaN	NaN	23.74	24.24	25.84	25.7
A0A452FR95	CFB	Uncharac	23.92	24.16	NaN	22.95	23.74	24.4	23.6	23.96
A0A452DPV2	LOC1021	NAD(P)(+	NaN	21.51	NaN	NaN	23.79	23.64	22.64	21.96
A0A452FJ48	RRAS2	RAS rela	25.54	25.32	NaN	NaN	23.81	24.28	24.78	24.57
A0A452E3Y8	TMC4	Transmem	26.93	25.79	NaN	NaN	23.84	23.55	24.55	24.63
A0A452G5W9	ALDH3B1	Aldehyde	25.79	25.6	NaN	NaN	23.94	24.41	25.49	25.18
A0A452FT54	GIPC2	GIPC PDZ	27.64	27.45	NaN	NaN	24.05	24.62	26.23	26.28
A0A452FGW9	GPAT4	Glycerol	25.95	25.99	NaN	NaN	24.28	24.4	25.67	25.43
A0A452G1U1	PSEN1	Presenil	25.97	25.74	NaN	NaN	24.87	NaN	25.21	25.45
P68215	FGA	Fibrinog	NaN	NaN	NaN	NaN	25.01	24.83	NaN	NaN
A0A452EAZ8	NDRG2	NDRG fam	27.44	27.39	NaN	NaN	25.27	25.46	26.83	26.72
A0A452FNL9	LYN	Tyrosine	26.32	26.4	NaN	NaN	25.42	25.63	26.1	26.09
A0A452FNC1	FAF2	Fas asso	27.48	27.5	NaN	NaN	25.53	25.56	26.81	26.56
A0A452EW11	C7	Compleme	26.87	26.68	NaN	NaN	25.66	25.43	27.28	27.25
A0A452G282	GNG7	Guanine	26.42	26.2	NaN	25.03	25.91	NaN	25.06	24.88
A0A452EAH5	ALPL	Alkaline	NaN	24.74	NaN	21.64	26.03	25.98	24.75	25.42
A0A452DYG7	LOC1021	BPI1 dom	NaN	NaN	NaN	NaN	26.31	26.49	23.14	23.58
A0A452E2C0	TTR	Transthy	NaN	NaN	NaN	21.86	26.37	26.91	NaN	23.99
U5S0D4	Lifr	Leukemia	22.45	22.4	NaN	NaN	NaN	NaN	NaN	NaN
Q9BG81	SCD1	Acyl-CoA	26.62	26.4	NaN	NaN	NaN	NaN	23.9	24.23
H9TKZ0	tram1	Transloc	24.34	24.69	NaN	NaN	NaN	NaN	NaN	NaN
A0A452FQY0	CYP20A1	Cytochro	25.89	26.73	NaN	NaN	NaN	NaN	25.31	23.17
A0A452E2N1	CYBRD1	Cytochro	24.78	24.54	NaN	21.74	NaN	NaN	23.66	22.93
A0A452FJ43	KDELR1	ER lumen	24.39	24.25	NaN	NaN	NaN	NaN	22.5	21.81
A0A452FNZ7	CAPN1	Uncharac	22.84	22.44	NaN	NaN	NaN	NaN	22.49	NaN
Q29430	N/A	Lysozyme	24.25	24.16	NaN	NaN	NaN	NaN	NaN	NaN
B9VR86	PSMA6	Proteaso	24.88	24.82	NaN	NaN	NaN	NaN	NaN	NaN
A0A452GBM7	ERP44	Endoplas	25.53	25.38	NaN	NaN	NaN	NaN	NaN	23.23
A0A452GBJ0	PDCD6IP	BRO1 dom	23.28	23.39	NaN	NaN	NaN	NaN	23.42	23.4
A0A452GB99	TAGLN2	Transgel	NaN	NaN	NaN	NaN	NaN	NaN	23.07	22.93
A0A452GAU5	LOC1021	Uncharac	24.98	24.78	NaN	NaN	NaN	NaN	25.14	25.03
A0A452GAH2	CS	Citrate	22.33	22.35	NaN	NaN	NaN	NaN	NaN	NaN
A0A452GAF7	HM13	Histocom	24.86	24.95	NaN	NaN	NaN	NaN	NaN	NaN
A0A452GA70	ADA	Adenosin	NaN	NaN	NaN	NaN	NaN	NaN	23.54	22.99
A0A452G9Y1	LOC1086	SERPIN d	23.68	23.54	NaN	NaN	NaN	NaN	NaN	NaN
A0A452G967	OSBPL9	Oxystero	20.71	20.8	NaN	NaN	NaN	NaN	NaN	NaN
A0A452G8R5	SCAMP4	Secretor	23.06	22.94	NaN	NaN	NaN	NaN	21.62	21.5
A0A452G8B2	CYB5B	Cytochro	25.47	25.6	NaN	22.91	NaN	NaN	22.61	NaN
A0A452G7V1	BSCL2	BSCL2 li	28.19	28.32	NaN	NaN	NaN	NaN	27.08	27.1

A0A452G7N6	S100A16	Protein	24.88	25.03	NaN	NaN	NaN	NaN	23.49	23.93
A0A452G7G7	TMCO1	Calcium	24.61	24.42	NaN	NaN	NaN	NaN	NaN	21.93
A0A452G789	IMMT	MICOS cc	21.96	21.98	NaN	NaN	NaN	NaN	NaN	NaN
A0A452G776	SRP54	Signal r	23.53	23.21	NaN	NaN	NaN	NaN	NaN	NaN
A0A452G6T9	PSMD14	MPN doma	23.93	23.42	NaN	NaN	NaN	NaN	NaN	NaN
A0A452G684	EMC1	ER membr	23.5	23.7	NaN	NaN	NaN	NaN	NaN	NaN
A0A452G5K1	PACC1	Proton a	22.86	22.9	NaN	NaN	NaN	NaN	NaN	NaN
A0A452G5I4	ARCN1	Coatomer	22.2	22.22	NaN	NaN	NaN	NaN	NaN	NaN
A0A452G5G8	DNM2	Dynamin	21.86	21.77	NaN	NaN	NaN	NaN	NaN	NaN
A0A452G5E3	ATP5PD	ATP synt	25.42	25.08	NaN	NaN	NaN	NaN	NaN	24.48
A0A452G572	CLTC	Clathrin	26.71	26.56	NaN	NaN	NaN	NaN	NaN	NaN
A0A452G510	VPS35	Vacuolar	22.89	22.96	NaN	NaN	NaN	NaN	21.81	21.39
A0A452G4T1	CEMIP2	Cell mig	23.17	23.44	NaN	NaN	NaN	NaN	22.94	NaN
A0A452G4D6	CCT2	Chaperon	24.68	26.54	NaN	NaN	NaN	NaN	NaN	22.31
A0A452G4A6	PCMT1	Protein-	22.56	22.75	NaN	NaN	NaN	NaN	22.4	NaN
A0A452G478	N/A	UBIQUIT]	25.97	26.13	NaN	NaN	NaN	NaN	NaN	NaN
A0A452G471	MARVELD	MARVEL c	23.91	23.4	NaN	NaN	NaN	NaN	NaN	NaN
A0A452G3Q4	RPL18A	60S ribc	26.59	26.81	NaN	NaN	NaN	NaN	NaN	NaN
A0A452G300	PSMD12	Proteasc	22.23	22.04	NaN	NaN	NaN	NaN	NaN	NaN
A0A452G2Z1	RPL3	Uncharac	24.84	28.37	NaN	NaN	NaN	NaN	21.67	21.88
A0A452G2T5	DYNC1I2	Dynein c	23.29	23.43	NaN	NaN	NaN	NaN	NaN	NaN
A0A452G2S0	EEF1E1	GST C-te	25.69	25.67	NaN	NaN	NaN	NaN	24.42	23.87
A0A452G2Q3	GLUL	Glutamir	23.95	24.22	NaN	NaN	NaN	NaN	NaN	NaN
A0A452G237	ATP2A2	Calcium-	23.9	23.64	NaN	NaN	NaN	NaN	NaN	NaN
A0A452G1Z7	SQLE	Squalene	24.54	24.42	NaN	NaN	NaN	NaN	NaN	NaN
A0A452G1Q5	RPL10	Ribosoma	27.08	26.84	NaN	NaN	NaN	NaN	NaN	NaN
A0A452G0Z5	PFKP	6-phosph	23.07	22.97	NaN	NaN	NaN	NaN	NaN	NaN
A0A452G0P9	PARVA	Parvin a	23.57	23.66	NaN	NaN	NaN	NaN	NaN	NaN
A0A452G069	WDR1	WD repea	25.42	25.11	NaN	22.74	NaN	NaN	23.73	NaN
A0A452G058	ATP13A3	Cation-t	22.82	22.71	NaN	NaN	NaN	NaN	21.81	NaN
A0A452FZZ9	LOC1021	Eukaryot	24.89	24.89	NaN	NaN	NaN	NaN	NaN	NaN
A0A452FZT8	SYNCRIP	Synaptot	24.79	25.21	NaN	NaN	NaN	NaN	NaN	NaN
A0A452FZR8	HTATIP2	HIV-1 Ta	NaN	22.66	NaN	NaN	NaN	NaN	NaN	22.07
A0A452FZL7	ADGRF1	Adhesior	22.34	NaN	NaN	NaN	NaN	NaN	21.92	NaN
A0A452FZ94	LOC1021	Uncharac	25.56	25.53	NaN	NaN	NaN	NaN	24.78	24.5
A0A452FZ49	STT3A	STT3 oli	26.11	26.92	NaN	NaN	NaN	NaN	23.01	23.18
A0A452FYW6	DYNC1H1	Dynein c	23.82	23.9	NaN	NaN	NaN	NaN	NaN	NaN
A0A452FYU0	TLL1	Metalloe	25.8	25.78	NaN	NaN	NaN	NaN	25.31	25.63
A0A452FYD0	ARL1	ADP ribc	23.79	23.54	NaN	NaN	NaN	NaN	NaN	NaN
A0A452FXS3	DPM1	Dolichol	23.85	23.65	NaN	NaN	NaN	NaN	NaN	NaN
A0A452FXN5	PAFAH1B	Platelet	24.33	24.39	NaN	NaN	NaN	NaN	NaN	NaN
A0A452FXK1	TOM1	Target c	21.97	22.6	NaN	NaN	NaN	NaN	NaN	NaN
A0A452FXJ0	LOC1021	RING-typ	NaN	22.79	NaN	NaN	NaN	NaN	NaN	20.95
A0A452FXD6	CLIC1	Chloride	21.04	20.88	NaN	NaN	NaN	NaN	23.78	23.28
A0A452FWV7	N/A	Pribosyl	23.74	24.17	NaN	NaN	NaN	NaN	NaN	NaN
A0A452FWR4	RBBP9	RB bindi	23.22	22.11	NaN	NaN	NaN	NaN	NaN	21.46
A0A452FWQ6	OLA1	Obg-like	23.06	22.7	NaN	NaN	NaN	NaN	NaN	NaN
A0A452FVS4	ACSS2	Acyl-CoA	23.28	23.47	NaN	NaN	NaN	NaN	NaN	NaN
A0A452FVM4	WARS	Tryptoph	28.06	27.95	NaN	NaN	NaN	20.36	24.54	24.37
A0A452FVJ4	SYVN1	Synoviol	23.02	22.43	NaN	NaN	NaN	NaN	NaN	NaN

A0A452FVJ2	PABPC1	Polyader	24.39	24.65	NaN	NaN	NaN	NaN	NaN	NaN
A0A452FVB0	ARPC3	Actin-re	23.78	23.3	NaN	22.1	NaN	NaN	22.45	22.07
A0A452FU58	ATP1B1	Sodium/p	25.09	25.13	NaN	NaN	NaN	NaN	23.3	23.61
A0A452FU56	N/A	Flavodox	25.43	25.57	NaN	NaN	NaN	NaN	NaN	21.26
A0A452FU33	SND1	Staphylc	27.76	25.72	NaN	NaN	NaN	NaN	22.96	NaN
A0A452FTX0	MPP5	Membrane	22.09	21.79	NaN	NaN	NaN	NaN	NaN	NaN
A0A452FTU0	MBLAC2	Metallo-	24.92	24.34	NaN	NaN	NaN	NaN	24.73	NaN
A0A452FTN4	PSAT1	Phosphos	23.66	23.93	NaN	NaN	NaN	NaN	23.08	22.71
A0A452FTH7	LOC1021	Anaphyla	26.98	26.88	NaN	NaN	NaN	NaN	25.17	25.4
A0A452FTB6	DNAJA2	Uncharac	25.19	24.62	NaN	NaN	NaN	NaN	23.36	23.59
A0A452FSW6	ESYT1	Extendec	22.6	22.19	NaN	NaN	NaN	NaN	NaN	NaN
A0A452FSN3	BMP1	Metalloe	24.16	24	NaN	NaN	NaN	NaN	23.97	23.9
A0A452FSI1	MESD	Mesoderm	NaN	21.64	NaN	21.83	NaN	NaN	NaN	NaN
A0A452FSB2	MYO1C	Myosin I	22.08	21.93	NaN	NaN	NaN	NaN	NaN	NaN
A0A452FRS6	ATP6V0C	V-type p	23.01	23.05	NaN	NaN	NaN	NaN	NaN	NaN
A0A452FRQ4	PON2	Paraoxor	22.96	23.08	NaN	NaN	NaN	NaN	20.81	NaN
A0A452FRI5	SH3YL1	SH3 and	21.8	21.46	NaN	NaN	NaN	20.52	21.37	21.57
A0A452FRG8	LOC1021	ANK_REP_	NaN	21.09	NaN	NaN	NaN	NaN	20.41	21.28
A0A452FR51	CRB3	Crumbs c	22.6	22.77	NaN	NaN	NaN	NaN	NaN	21.58
A0A452FQY9		AP compl	22.38	21.91	NaN	NaN	NaN	NaN	NaN	NaN
A0A452FQV1	CCT7	T-comple	25.88	25.87	NaN	NaN	NaN	NaN	20.59	20.58
A0A452FQM7	ATP6V1E	ATPase H	24.16	24.31	NaN	22.96	NaN	NaN	23.91	23.9
A0A452FPF6	FARSA	Phenylal	22.96	22.78	NaN	NaN	NaN	NaN	NaN	NaN
A0A452FPD6	SEL1L	SEL1L ac	27.09	26.7	NaN	NaN	NaN	NaN	23.8	23.07
A0A452FNW0	EEF1G	Uncharac	26.96	26.8	NaN	NaN	NaN	NaN	NaN	NaN
A0A452FNT7	ADPGK	ADP depe	24.25	23.78	NaN	NaN	NaN	NaN	22.54	22.68
A0A452FNH4	ATL3	Atlastir	21.87	23.39	NaN	NaN	NaN	NaN	NaN	NaN
A0A452FNE1	CTDSP1	CTD smal	22.35	22.59	NaN	NaN	NaN	NaN	NaN	NaN
A0A452FMD3	COPB2	Coatomer	23.66	23.96	NaN	NaN	NaN	NaN	NaN	NaN
A0A452FLY2	ZDHHC14	Palmitoy	22.91	23.7	NaN	NaN	NaN	21.92	22.56	NaN
A0A452FLQ8	EIF4A1	Eukaryot	23.22	22.49	NaN	NaN	NaN	NaN	NaN	NaN
A0A452FLK7	ATP5PF	ATP synt	23.04	23.08	NaN	NaN	NaN	NaN	22.56	22.63
A0A452FLJ7	GFPT1	Glutamir	23.56	22.63	NaN	NaN	NaN	21.47	21.94	NaN
A0A452FK79	ATP5MF	Uncharac	24.41	24.24	NaN	NaN	NaN	NaN	NaN	NaN
A0A452FK08	BLVRB	Biliverc	22.98	22.52	NaN	NaN	NaN	NaN	22.6	22.81
A0A452FJN9	YIF1A	Yip1 int	23.05	23.17	NaN	NaN	NaN	NaN	NaN	NaN
A0A452FJF7	EIF3I	Eukaryot	26.09	26.07	NaN	NaN	NaN	NaN	NaN	22.18
A0A452FJ88	SCAMP2	Secretor	26.22	26.05	NaN	NaN	NaN	NaN	25.58	25.13
A0A452FJ27	DCTN2	Dynactir	25.88	26.02	NaN	NaN	NaN	NaN	24.89	24.83
A0A452FIZ8	EXOC5	Exocyst	23.72	23.51	NaN	NaN	NaN	NaN	23.48	23.61
A0A452FIX2	CYB561	Cytochrc	23	NaN	NaN	NaN	NaN	NaN	22.49	22.94
A0A452FIW1	CCDC47	Coiled-c	23.63	24.03	NaN	NaN	NaN	NaN	NaN	NaN
A0A452FIH4	TXNL1	Thioredc	23.83	23.94	NaN	NaN	NaN	NaN	22.36	22.64
A0A452FIG7	ESD	S-formyl	23.02	23.69	NaN	NaN	NaN	NaN	NaN	NaN
A0A452FIA8	SEC63	SEC63 hc	23.82	23.99	NaN	NaN	NaN	NaN	NaN	NaN
A0A452FI28	TBCA	Tubulin-	23.36	22.83	NaN	NaN	NaN	NaN	22.54	22.9
A0A452FI11	ERLIN1	ER lipic	23.83	23.51	NaN	NaN	NaN	NaN	NaN	NaN
A0A452FHY0	AQP3	Aquapori	25.14	25.63	NaN	NaN	NaN	NaN	NaN	NaN
A0A452FHU8	CCDC91	Coiled-c	20.28	21.78	NaN	NaN	NaN	NaN	NaN	NaN
A0A452FHM9	SEPTIN1	Septin 1	23.4	22.28	NaN	NaN	NaN	NaN	NaN	NaN

A0A452FHI0	LOC1021	Methyltr	25.34	24.99	NaN	NaN	NaN	NaN	22.73	23.42
A0A452FGQ3	ARPC1B	Actin-re	22.36	22.62	NaN	NaN	NaN	NaN	NaN	NaN
A0A452FGI6	NME3	Nucleosi	21.21	21.47	NaN	NaN	NaN	NaN	NaN	21.68
A0A452FGH0	TINAGL1	Tubuloir	24.52	24.38	NaN	NaN	NaN	NaN	23.55	23.51
A0A452FFD7	CFH	Uncharac	23.96	23.94	NaN	NaN	NaN	NaN	22.38	22.63
A0A452FF82	EMC2	ER membr	24.25	23.16	NaN	NaN	NaN	NaN	NaN	NaN
A0A452FEY1	EIF2B2	Eukaryot	22.41	22.37	NaN	NaN	NaN	NaN	NaN	NaN
A0A452FEX1	PLPP1	Phosphol	23.67	23.16	NaN	NaN	NaN	NaN	23.39	23.3
A0A452FEQ8	UBTD1	Ubiquiti	23.96	23.49	NaN	NaN	NaN	NaN	23.38	23.2
A0A452FEP6	SEC62	SEC62 hc	24.69	25	NaN	NaN	NaN	NaN	NaN	NaN
A0A452FEN4	GPAM	Glycerol	23.12	23.41	NaN	NaN	NaN	NaN	22.24	22.11
A0A452FEM7	PRKAG1	Protein	22.57	22.86	NaN	NaN	NaN	NaN	NaN	NaN
A0A452FEH4	EIF3F	Eukaryot	24.96	25.49	NaN	NaN	NaN	NaN	NaN	NaN
A0A452FDS2	PSMC5	Proteasc	23.92	23.61	NaN	NaN	NaN	NaN	NaN	NaN
A0A452FDQ1	TECR	Trans-2,	23.6	23.64	NaN	NaN	NaN	NaN	NaN	NaN
A0A452FDJ3	RAB33B	RAB33B,	22.76	22.3	NaN	NaN	NaN	NaN	22.46	22.19
A0A452FCU2	FDPS	Farnesyl	25.1	25.06	NaN	NaN	NaN	NaN	NaN	NaN
A0A452FBM2	HSPA9	Heat shc	21.75	21.41	NaN	NaN	NaN	NaN	22.08	21.68
A0A452FBI7	PCBP2	Poly(rC)	21.74	22.05	NaN	NaN	NaN	NaN	NaN	NaN
A0A452FB58	SLC25A1	Solute c	22.54	22.3	NaN	NaN	NaN	NaN	NaN	NaN
A0A452FB30	SRPRB	SRP rece	26.14	26.1	NaN	NaN	NaN	NaN	NaN	NaN
A0A452FB11	PSMA1	Proteasc	24.97	25.31	NaN	NaN	NaN	NaN	23.69	23.4
A0A452FAZ1	PSMB4	Proteasc	25.75	25.52	NaN	NaN	NaN	NaN	NaN	NaN
A0A452FAX2	LASP1	LIM and	23.04	23.08	NaN	NaN	NaN	NaN	22.88	NaN
A0A452FAS9	SUCLG2	Succinat	23.39	23.44	NaN	NaN	NaN	NaN	NaN	NaN
A0A452FAQ2	N/A	Uncharac	26.63	26.56	NaN	NaN	NaN	NaN	NaN	NaN
A0A452FA09	MUC20	Uncharac	21.93	21.6	NaN	NaN	NaN	NaN	23.55	23.48
A0A452F9S2	LOC1021	Ig-like	21.73	21.85	NaN	NaN	NaN	NaN	NaN	NaN
A0A452F8M5	COPE	Coatomer	22.56	22.06	NaN	NaN	NaN	NaN	NaN	NaN
A0A452F8I1	MPDU1	Mannose-	25.22	24.2	NaN	NaN	NaN	NaN	23.27	23.1
A0A452F8F4	AIMP1	tRNA-bir	25.26	25.04	NaN	NaN	NaN	NaN	NaN	22.73
A0A452F7R9	TM4SF18	Transmem	26.72	26.57	NaN	23.55	NaN	NaN	24.66	25.05
A0A452F7N8	AHCYL2	Adenosyl	23.59	23.32	NaN	NaN	NaN	NaN	NaN	NaN
A0A452F719	MANF	Mesencep	NaN	NaN	NaN	23.08	NaN	NaN	22.55	22.65
A0A452F711	FKBP3	Peptidyl	24.06	22.23	NaN	21.44	NaN	NaN	NaN	21.41
A0A452F6Y2	N/A	Ig-like	24.35	24.22	NaN	NaN	NaN	20.51	23.85	NaN
A0A452F6I3	PGM1	Phosphog	23.29	23.72	NaN	NaN	NaN	NaN	23.14	NaN
A0A452F5Z8	SPPL2B	Signal p	23.78	22.81	NaN	NaN	NaN	NaN	22.4	22.02
A0A452F5W5	SNAP29	Synaptos	25.7	25.81	NaN	NaN	NaN	NaN	24.79	25.2
A0A452F5W4	APRT	Adenine	23.84	NaN	NaN	20.29	NaN	NaN	NaN	NaN
A0A452F5I1	RTRAF	RNA trar	22.23	22.58	NaN	NaN	NaN	NaN	NaN	NaN
A0A452F5D1	SLC1A1	Solute c	29.21	29.91	NaN	NaN	NaN	25.76	28.47	28.29
A0A452F561	KRTCAP2	Keratinc	22.75	23.22	NaN	NaN	NaN	NaN	20.31	NaN
A0A452F550	PSMD7	Proteasc	25.34	25.02	NaN	NaN	NaN	NaN	NaN	NaN
A0A452F4B6	VCP	Valosin	27.53	27.69	NaN	NaN	NaN	NaN	20.64	21.64
A0A452F429	UCHL3	Ubiquiti	23.63	23.25	NaN	NaN	NaN	NaN	NaN	NaN
A0A452F420	FLOT2	Flotilli	25.2	25.13	NaN	23.82	NaN	NaN	24.13	24.28
A0A452F3U3	CCT5	Chaperor	21.54	22.37	NaN	NaN	NaN	NaN	NaN	NaN
A0A452F3P2	TMEM222	Transmem	22.53	22.79	NaN	NaN	NaN	NaN	NaN	NaN
A0A452F2Z5	GOLPH3L	Uncharac	24.61	25.26	NaN	NaN	NaN	NaN	24.29	22.99

A0A452F2U6	DNAJB6	J domair	20.62	23.03	NaN	NaN	NaN	NaN	NaN	NaN
A0A452F2M7	NDUFB10	NADH:ubi	23.09	23.29	NaN	21.17	NaN	NaN	NaN	NaN
A0A452F2M3	LOC1021	Uncharac	22.35	23.37	NaN	NaN	NaN	NaN	NaN	NaN
A0A452F2J4	S100A10	S100 cal	22.77	23.23	NaN	NaN	NaN	NaN	NaN	NaN
A0A452F2I2	EPHX1	Epoxide	28.29	28.5	NaN	NaN	NaN	NaN	25.71	25.69
A0A452F1Y8	SLC39A9	Solute c	21.81	21.1	NaN	NaN	NaN	NaN	NaN	NaN
A0A452F1R7	ST13	TPR_REGI	24.76	24.69	NaN	NaN	NaN	NaN	22.84	23.04
A0A452F1P5	RPS28	Uncharac	27.68	27.45	NaN	NaN	NaN	NaN	NaN	NaN
A0A452F130	VPS37B	VPS37B s	23.03	22.73	NaN	NaN	NaN	NaN	22.55	22.4
A0A452F0Q1	N/A	Uncharac	27.39	27.39	NaN	NaN	NaN	NaN	NaN	NaN
A0A452F0K0	SRP19	Signal r	22.68	23.32	NaN	NaN	NaN	NaN	NaN	NaN
A0A452F0J6	UGGT1	UDP-gluc	26.15	26.37	NaN	NaN	NaN	NaN	22.95	21.96
A0A452F0I5	ATP1A1	Sodium/p	26.88	26.29	NaN	NaN	NaN	NaN	22.76	23.3
A0A452F0G8	LTA4H	Leukotri	23.92	23.98	NaN	NaN	NaN	NaN	22.55	NaN
A0A452F0G5	EIF5A2	Eukaryot	26.58	26.98	NaN	NaN	NaN	23.42	24.96	25.21
A0A452F0G2	HSD17B1	Hydroxys	25.38	25.74	NaN	21.99	NaN	NaN	23.34	NaN
A0A452F008	GMPPA	GDP-manr	23.81	23.74	NaN	NaN	NaN	NaN	NaN	NaN
A0A452F004	C1QB	Compleme	23.77	23.16	NaN	NaN	NaN	NaN	23.54	NaN
A0A452EZZ3	DAD1	Dolichyl	25.55	25.31	NaN	NaN	NaN	NaN	22.44	22.59
A0A452EZ21	PSMD1	26S prot	23.55	23.61	NaN	NaN	NaN	NaN	NaN	NaN
A0A452EYI5	MED21	Mediator	23.2	23.27	NaN	NaN	NaN	NaN	23.19	21.92
A0A452EY73	PSMD2	26S prot	24.41	23.64	NaN	NaN	NaN	NaN	NaN	NaN
A0A452EY25		Glycyl-t	21.69	21.49	NaN	NaN	NaN	NaN	NaN	NaN
A0A452EXP6	RPL30	Ribosoma	25.98	26.82	NaN	NaN	NaN	NaN	23.94	NaN
A0A452EXG6	NAP1L4	Nucleosc	22.8	23.47	NaN	NaN	NaN	NaN	22.01	NaN
A0A452EXC9	ATP6V1B	Vacuolar	23.22	23.07	NaN	NaN	NaN	NaN	NaN	NaN
A0A452EWR4	DHRSX	Dehydrog	22.86	22.29	NaN	NaN	NaN	NaN	NaN	23.38
A0A452EW60	LOC1021	Uncharac	24.37	24.72	NaN	NaN	NaN	23.43	24.3	23.88
A0A452EVX4	TCP1	T-comple	25.4	25.43	NaN	NaN	NaN	NaN	NaN	NaN
A0A452EUK4	PPA1	Pyrophos	27.32	27.25	NaN	NaN	NaN	NaN	23.71	23.89
A0A452EUB1	INPP5A	Inositol	25.05	24.79	NaN	NaN	NaN	NaN	24.44	24.67
A0A452EUA2	RAB27B	RAB27B,	22.13	21.91	NaN	NaN	NaN	NaN	NaN	NaN
A0A452EU44	YWHAH	Tyrosine	23	22.73	NaN	NaN	NaN	NaN	22.38	22.51
A0A452EU23	LIPG	Lipase (	24.59	24.24	NaN	NaN	NaN	NaN	24.6	24.64
A0A452ETZ6	ACTN1	Actinin	24.56	24.62	NaN	NaN	NaN	NaN	NaN	NaN
A0A452ETY1	LOC1021	Uncharac	24.7	24.63	NaN	NaN	NaN	NaN	NaN	NaN
A0A452ETR0	CMTM8	CKLF lik	26.36	26.16	NaN	NaN	NaN	NaN	24.54	24.61
A0A452ETA7	CLTA	Clathrir	24.19	23.88	NaN	NaN	NaN	NaN	NaN	NaN
A0A452ET94	NDRG1	N-myc dc	24.3	24.22	NaN	NaN	NaN	NaN	NaN	NaN
A0A452ET01	PSMB7	Proteasc	23.86	23.85	NaN	NaN	NaN	NaN	NaN	NaN
A0A452ESS4	PGM2	Phosphog	21.18	20.9	NaN	NaN	NaN	NaN	NaN	NaN
A0A452ESI0	EMC10	ER membr	22.52	23.06	NaN	NaN	NaN	NaN	NaN	NaN
A0A452ESG4	RACK1	Receptor	27.78	27.74	NaN	22.9	NaN	NaN	NaN	NaN
A0A452ES44	TGFBR3	Transfor	24.34	24.23	NaN	NaN	NaN	NaN	24.09	24.31
A0A452ERM1	GNG10	Guanine	25.39	NaN	NaN	NaN	NaN	NaN	24.68	23.81
A0A452ERF6	C19orf2	Chromosc	24.37	NaN	NaN	NaN	NaN	NaN	23.98	NaN
A0A452ERB9	GAS6	Growth a	28.31	27.42	NaN	24.83	NaN	NaN	27.49	27.29
A0A452ER37	PSMC2	Proteasc	23.37	23.64	NaN	NaN	NaN	NaN	NaN	NaN
A0A452EQY7	F5	Coagulat	22.78	23.01	NaN	NaN	NaN	NaN	NaN	NaN
A0A452EQY2	DDOST	Dolichyl	28.99	28.26	NaN	23.71	NaN	NaN	22.52	23.18

A0A452EQF0	UQCRFS1	Cytochrc	24.02	23.48	NaN	19.48	NaN	NaN	NaN	NaN
A0A452EQE5	UQCRC1	Ubiquinc	24.57	24.31	NaN	NaN	NaN	NaN	NaN	23.23
A0A452EQ53	FKBP11	Peptidyl	27.26	27.04	NaN	21.49	NaN	NaN	24.21	24.26
A0A452EQ13	RPL26L1	Ribosoma	20.78	21.69	NaN	NaN	NaN	NaN	NaN	NaN
A0A452EPW9	N/A	Uncharac	24.34	24.21	NaN	NaN	NaN	NaN	NaN	NaN
A0A452EP42	HHIPL2	HHIP lik	22.7	22.89	NaN	NaN	NaN	NaN	23.17	NaN
A0A452ENS6	ABCC3	ATP binc	23.98	24.26	NaN	NaN	NaN	NaN	NaN	NaN
A0A452ENK4	MYO1D	Myosin I	22.18	22.09	NaN	NaN	NaN	NaN	NaN	NaN
A0A452ENE9	C2orf72	Chromosc	22.66	22.35	NaN	NaN	NaN	NaN	21.81	21.64
A0A452ENC0	SEC61G	SEC61 tr	23.75	23.45	NaN	NaN	NaN	NaN	NaN	NaN
A0A452EN77	PTPRF	Protein	25.66	25.68	NaN	NaN	NaN	NaN	NaN	NaN
A0A452EN40	DHCR7	7-dehydr	21.89	22.03	NaN	NaN	NaN	NaN	NaN	NaN
A0A452EMM4	TMEM120	Transmem	25.18	24.81	NaN	NaN	NaN	NaN	NaN	NaN
A0A452EM22	TC2N	Tandem C	21.96	22.35	NaN	NaN	NaN	NaN	22.19	21.85
A0A452ELK2	ACTR1A	Actin re	26.75	26.22	NaN	NaN	NaN	NaN	23.12	NaN
A0A452ELB8	LIFR	LIF rece	23.97	24.16	NaN	NaN	NaN	NaN	23.34	22.08
A0A452EL27	EIF3L	Eukaryot	24.57	24.3	NaN	NaN	NaN	NaN	NaN	NaN
A0A452EKZ5	ZMPSTE2	CAAX pre	21.52	21.7	NaN	NaN	NaN	NaN	NaN	NaN
A0A452EK79	TMED7	GOLD dom	25.41	25.32	NaN	NaN	NaN	NaN	22.71	22.9
A0A452EIV1	SEPTIN7	Septin 7	23.08	23.28	NaN	NaN	NaN	NaN	NaN	NaN
A0A452EIP8	PTBP3	Polypyri	21.56	23.33	NaN	NaN	NaN	NaN	NaN	NaN
A0A452EI06	CAPZA2	F-actin-	24.41	25.32	NaN	23.32	NaN	NaN	NaN	23.61
A0A452EHR0	TARS	Threonyl	25.17	24.91	NaN	NaN	NaN	NaN	NaN	NaN
A0A452EHN1	PCOLCE	Procolla	NaN	NaN	NaN	NaN	NaN	20.01	21.93	21.8
A0A452EHJ6	HADH	Uncharac	25.04	25.38	NaN	NaN	NaN	NaN	24.1	23.59
A0A452EH52	SULT1C4	Sulfotra	22.06	22.47	NaN	NaN	NaN	NaN	22.09	21.86
A0A452EGY3	RPS21	40S ribc	26.97	27.08	NaN	NaN	NaN	NaN	23.37	NaN
A0A452EGI2	STT3B	STT3 oli	23.19	22.98	NaN	NaN	NaN	NaN	NaN	NaN
A0A452EGC2	VDAC2	Voltage	23.94	24.53	NaN	NaN	NaN	NaN	NaN	23.83
A0A452EG75	MGST2	Microsom	23.7	23.35	NaN	NaN	NaN	NaN	NaN	22.41
A0A452EFG2	TOR1AIP	Torsin 1	22.71	23.28	NaN	21.19	NaN	NaN	23.68	23.55
A0A452EFA5	APMAP	Adipocyt	25.52	25.38	NaN	20.96	NaN	NaN	23.72	23.35
A0A452EEZ2	OSTF1	Osteocla	NaN	NaN	NaN	NaN	NaN	NaN	22.66	22.15
A0A452EEM0	RPS27L	40S ribc	25.18	24.87	NaN	NaN	NaN	NaN	NaN	NaN
A0A452EDZ8	DHDDS	Alkyl tr	24.6	24.66	NaN	NaN	NaN	NaN	NaN	NaN
A0A452ED96	TAX1BP3	Tax1-bir	24.64	24.64	NaN	NaN	NaN	NaN	NaN	22.32
A0A452ECX7	YWHAQ	14_3_3 c	25.24	25.1	NaN	NaN	NaN	21.92	22.9	23.86
A0A452ECW3	LOC1086	Uncharac	24.96	25.2	NaN	NaN	NaN	NaN	NaN	NaN
A0A452ECM3	LIN7C	Protein	22.69	23.08	NaN	NaN	NaN	NaN	NaN	NaN
A0A452EC92	SRPRA	SRP rece	24.39	24.27	NaN	NaN	NaN	NaN	NaN	22.64
A0A452EC85	PTX3	Pentraxi	24.3	23.6	NaN	NaN	NaN	NaN	23.68	23.78
A0A452EC68	ATP8B1	Phosphol	22.61	22.73	NaN	NaN	NaN	NaN	NaN	NaN
A0A452EC19	SSR3	Signal s	27.42	27.13	NaN	NaN	NaN	NaN	23.35	23.24
A0A452EBU5	KCTD12	Potassiu	21.87	NaN	NaN	21.23	NaN	NaN	NaN	NaN
A0A452EBT9	HID1	HID1 dom	24.35	24.39	NaN	NaN	NaN	NaN	NaN	NaN
A0A452EBE7	SDSL	Serine c	23.91	24.13	NaN	NaN	NaN	22.36	23	23.11
A0A452EB34	SLC25A6	Solute c	25.73	25.64	NaN	NaN	NaN	NaN	NaN	NaN
A0A452EB00	EIF3M	Eukaryot	22.53	22.35	NaN	NaN	NaN	NaN	NaN	NaN
A0A452EAX8	DPAGT1	Dolichyl	22.5	22.39	NaN	NaN	NaN	NaN	NaN	NaN
A0A452EA67	ERLEC1	Endoplas	25.07	25.43	NaN	NaN	NaN	NaN	23.66	24.01

A0A452E9W5	JAGN1	Jagunal	22.42	22.83	NaN	NaN	NaN	NaN	NaN	NaN
A0A452E9T3	N/A	Uncharac	24.24	24.1	NaN	22.13	NaN	NaN	23.91	23.55
A0A452E9F4	LOC1086	eIF2B_5	22.27	22.38	NaN	NaN	NaN	NaN	NaN	NaN
A0A452E9E7	RAP2B	RAP2B, n	25.68	26.52	NaN	22.66	NaN	23.55	25.82	26.34
A0A452E9C1	EIF3H	Eukaryot	23.3	23.4	NaN	NaN	NaN	NaN	NaN	NaN
A0A452E9A3	CLIC4	Chloride	NaN	23.23	NaN	NaN	NaN	NaN	23.08	23.83
A0A452E8T2	OLR1	Oxidized	NaN	NaN	NaN	NaN	NaN	NaN	21.91	21.64
A0A452E8N9	GPD1L	Glycerol	22.78	22.69	NaN	NaN	NaN	20.87	22.63	23.15
A0A452E813	PSMB5	Proteaso	23.26	23.35	NaN	NaN	NaN	NaN	NaN	NaN
A0A452E7W8	PON3	Paraoxor	24.91	25.4	NaN	NaN	NaN	NaN	22.86	23.06
A0A452E7S5	DKK3	Dickkopf	NaN	NaN	NaN	NaN	NaN	NaN	22.65	22.5
A0A452E7M2	IMPDH2	Inosine-	21.95	22.78	NaN	NaN	NaN	NaN	NaN	NaN
A0A452E7A0	PLG	Plasmino	25.74	NaN	NaN	NaN	NaN	NaN	26.97	26.82
A0A452E724	C1QC	Compleme	22.47	NaN	NaN	NaN	NaN	NaN	22.07	NaN
A0A452E701	VARS	Valyl-tR	23.08	22.44	NaN	NaN	NaN	NaN	NaN	NaN
A0A452E6T1	RPL32	Ribosoma	22.87	22.83	NaN	NaN	NaN	NaN	NaN	NaN
A0A452E6R6	N/A	Uncharac	26.34	26.24	NaN	NaN	NaN	NaN	26.58	26.68
A0A452E6J2	DARS	Aspartyl	25.07	25.03	NaN	NaN	NaN	NaN	NaN	NaN
A0A452E6F8	EIF2S1	Eukaryot	24.37	24.23	NaN	NaN	NaN	NaN	NaN	21.82
A0A452E5Q4	LOC1086	Mitochor	24.18	25.44	NaN	22.21	NaN	NaN	NaN	NaN
A0A452E5C6	CMTM6	CKLF lik	22.15	22.63	NaN	NaN	NaN	NaN	NaN	NaN
A0A452E5B3	LOC1086	Ig-like	24.09	24.48	NaN	NaN	NaN	NaN	22.79	23.26
A0A452E4F5	RNASE4	Ribonucl	25.95	25.76	NaN	NaN	NaN	NaN	NaN	24.73
A0A452E4E8	SPCS2	Signal p	26.13	26.13	NaN	NaN	NaN	NaN	23.95	NaN
A0A452E410	TMEM59	Transmem	21.41	NaN	NaN	NaN	NaN	NaN	23.31	23.69
A0A452E3Q9	TMEM125	Transmem	22.6	22.15	NaN	NaN	NaN	20.2	NaN	NaN
A0A452E3F8	TOR4A	Torsin f	23.52	23.28	NaN	NaN	NaN	NaN	24.01	23.81
A0A452E398	PIP4K2C	Phosphat	21.94	23.02	NaN	NaN	NaN	NaN	NaN	22.03
A0A452E356	PSMC3	Proteaso	25	24.91	NaN	NaN	NaN	NaN	NaN	NaN
A0A452E2D7	SEC61A1	SEC61 tr	27.79	26.67	NaN	21.29	NaN	NaN	23.67	23.5
A0A452E278	EIF3B	Eukaryot	23.85	23.9	NaN	NaN	NaN	NaN	NaN	NaN
A0A452E1Y6	TM9SF2	Transmem	23.54	23.53	NaN	NaN	NaN	NaN	22.82	22.54
A0A452E0V0	ETF1	eRF1_1 d	23.16	23.25	NaN	NaN	NaN	NaN	NaN	NaN
A0A452E0G5	EIF4E	Uncharac	21.31	21.79	NaN	NaN	NaN	NaN	NaN	NaN
A0A452E044	NIF3L1	NIF3-lik	22.82	23.16	NaN	NaN	NaN	NaN	NaN	NaN
A0A452DZV8	LRATD2	LRAT dom	25.54	25.27	NaN	NaN	NaN	NaN	24.15	23.65
A0A452DZK5	PSMA5	Proteaso	25.57	25.61	NaN	NaN	NaN	NaN	25.04	24.46
A0A452DZ71	GLOD4	Glyoxala	22.41	22.39	NaN	NaN	NaN	NaN	NaN	NaN
A0A452DZ39	CRK	CRK prot	23.69	23.93	NaN	NaN	NaN	NaN	NaN	NaN
A0A452DYV1	PA2G4	Peptidas	25.2	25.33	NaN	NaN	NaN	NaN	NaN	NaN
A0A452DXX8	ADGRF5	Adhesion	23.22	22.71	NaN	NaN	NaN	NaN	NaN	NaN
A0A452DXH7	PDXK	Pyridoxa	25.62	25.45	NaN	NaN	NaN	20.73	22.8	22.41
A0A452DX88	TMEM87A	Transmem	23.24	23.36	NaN	NaN	NaN	NaN	NaN	21.36
A0A452DWY1	VPS29	Vacuolar	23.56	23.46	NaN	NaN	NaN	NaN	NaN	NaN
A0A452DWS2	TSTA3	Tissue s	22.86	23.84	NaN	NaN	NaN	NaN	22.01	21.86
A0A452DWN7	NEDD8	NEDD8 ub	23.15	23.57	NaN	NaN	NaN	NaN	NaN	NaN
A0A452DW82	BCAT2	Branched	24.94	24.68	NaN	NaN	NaN	NaN	NaN	NaN
A0A452DW49	BCAT2	Branched	23.96	23.72	NaN	22.13	NaN	NaN	NaN	NaN
A0A452DVN5	RER1	Protein	21.98	21.92	NaN	NaN	NaN	NaN	NaN	NaN
A0A452DVD7	TMEM214	Transmem	NaN	21.16	NaN	21.11	NaN	NaN	NaN	NaN

A0A452DU54	TMEM205	Transmen	23.17	22.45	NaN	NaN	NaN	NaN	NaN	21.27
A0A452DU06	SNX2	Sorting	20.96	20.67	NaN	NaN	NaN	NaN	NaN	NaN
A0A452DU01	TPRG1L	Tumor pı	24.96	24.63	NaN	NaN	NaN	NaN	NaN	NaN
A0A452DTV6	RTN4	Reticulc	27.64	27.35	NaN	24.11	NaN	23.98	25.51	25.72
A0A452DTU9	CFI	Uncharac	20.47	NaN	NaN	NaN	NaN	NaN	22.24	22.4
A0A452DTI0	COMT	Catechol	27.29	27.04	NaN	NaN	NaN	NaN	NaN	NaN
A0A452DTE6	SEC11C	Signal p	25.83	26.07	NaN	NaN	NaN	NaN	NaN	NaN
A0A452DT65	LOC1086	GTP-binc	23.11	23.4	NaN	NaN	NaN	NaN	NaN	NaN
A0A452DT54	FRK	Tyrosine	23.05	23.33	NaN	NaN	NaN	NaN	NaN	22.42
A0A452DT40	ACTBL2	Actin be	NaN	29.86	NaN	NaN	NaN	NaN	NaN	28.07
A0A452DT26	PSMD13	PCI doma	24.09	24.01	NaN	NaN	NaN	NaN	NaN	NaN
A0A452DSJ6	SRP9	Signal ı	22.41	21.78	NaN	NaN	NaN	NaN	NaN	NaN
A0A452DSJ3	N/A	G_PROTEI	22.79	22.9	NaN	NaN	NaN	NaN	NaN	NaN
A0A452DRI6	IST1	IST1 fac	24.45	24.1	NaN	NaN	NaN	NaN	24.07	24.22
A0A452DRD5	EIF3E	Eukaryot	23.77	23.57	NaN	NaN	NaN	NaN	NaN	NaN
A0A452DR09	PNPO	Pyridoxa	23.46	23.11	NaN	NaN	NaN	NaN	NaN	NaN
A0A452DQ55	EIF2S3	Tr-type	24.67	24.48	NaN	NaN	NaN	NaN	21.68	NaN
A0A452DQ07	PSMD3	Proteasc	25.48	25.39	NaN	NaN	NaN	NaN	NaN	NaN
A0A452DPT3	GOLPH3	Golgi ph	23.96	24.55	NaN	NaN	NaN	NaN	24.11	23.61
A0A452DPL6	SNX3	Sorting	23.06	23.81	NaN	NaN	NaN	NaN	NaN	22.61
A0A452DP54	BIN2	BAR doma	22.33	NaN	NaN	NaN	NaN	NaN	21.46	21.44
A0A452DNI4	MAN2B1	Uncharac	NaN	23.18	NaN	NaN	NaN	NaN	26.01	25.2
A0A452DMZ0	DPM3	Dolichol	23.03	22.95	NaN	NaN	NaN	NaN	NaN	NaN
A0A452DMN3	SRM	Spermidi	22.38	22.54	NaN	NaN	NaN	NaN	22.78	NaN
A0A452DMM0	RPN2	Dolichyl	27.51	27.36	NaN	NaN	NaN	NaN	23.56	22.77
A0A452DM62	N/A	Reverse	21.96	21.79	NaN	NaN	NaN	NaN	21.89	22.24
A0A452DLZ6	SPCS1	Signal p	26.28	25.86	NaN	NaN	NaN	NaN	NaN	NaN
A0A452DLU5	SEC11A	Signal p	24.21	24.22	NaN	NaN	NaN	NaN	NaN	NaN
A0A452DLS5	ERGIC1	Endoplas	27.24	23.82	NaN	20.36	NaN	NaN	21.29	21.4
A0A452DL79	PRXL2A	Peroxire	23.83	23.89	NaN	NaN	NaN	NaN	NaN	NaN
A0A452DL52	PFKL	6-phosph	25.77	25.27	NaN	NaN	NaN	NaN	NaN	NaN
A0A452DL24	PGRMC1	Progeste	22.83	23.06	NaN	NaN	NaN	NaN	NaN	NaN
A0A452DKQ4	ATP11A	Phosphol	21.22	21.37	NaN	NaN	NaN	NaN	NaN	NaN
A0A452DK06	EDF1	Endothel	23.12	22.98	NaN	NaN	NaN	20.22	NaN	20.89
A0A452DJY2	VPS26A	VPS26, ı	25.83	25.9	NaN	NaN	NaN	NaN	NaN	NaN
A0A452DJQ9	AIFM1	Apoptosi	22.58	23.27	NaN	NaN	NaN	NaN	NaN	NaN
A0A0A7CPQ3	CRABP2	Cellulaı	24.7	24.65	NaN	NaN	NaN	NaN	24.03	24.23
