# Supplementary figures and images for: Changes in Caprine Milk Fat Globule Membrane Proteins after Heat Treatment Using a Label-Free Proteomics Technique
Source: Foods. 2022 Sep 5;11(17):2705. doi: 10.3390/foods11172705 (PMC9455663; doi:10.3390/foods11172705)

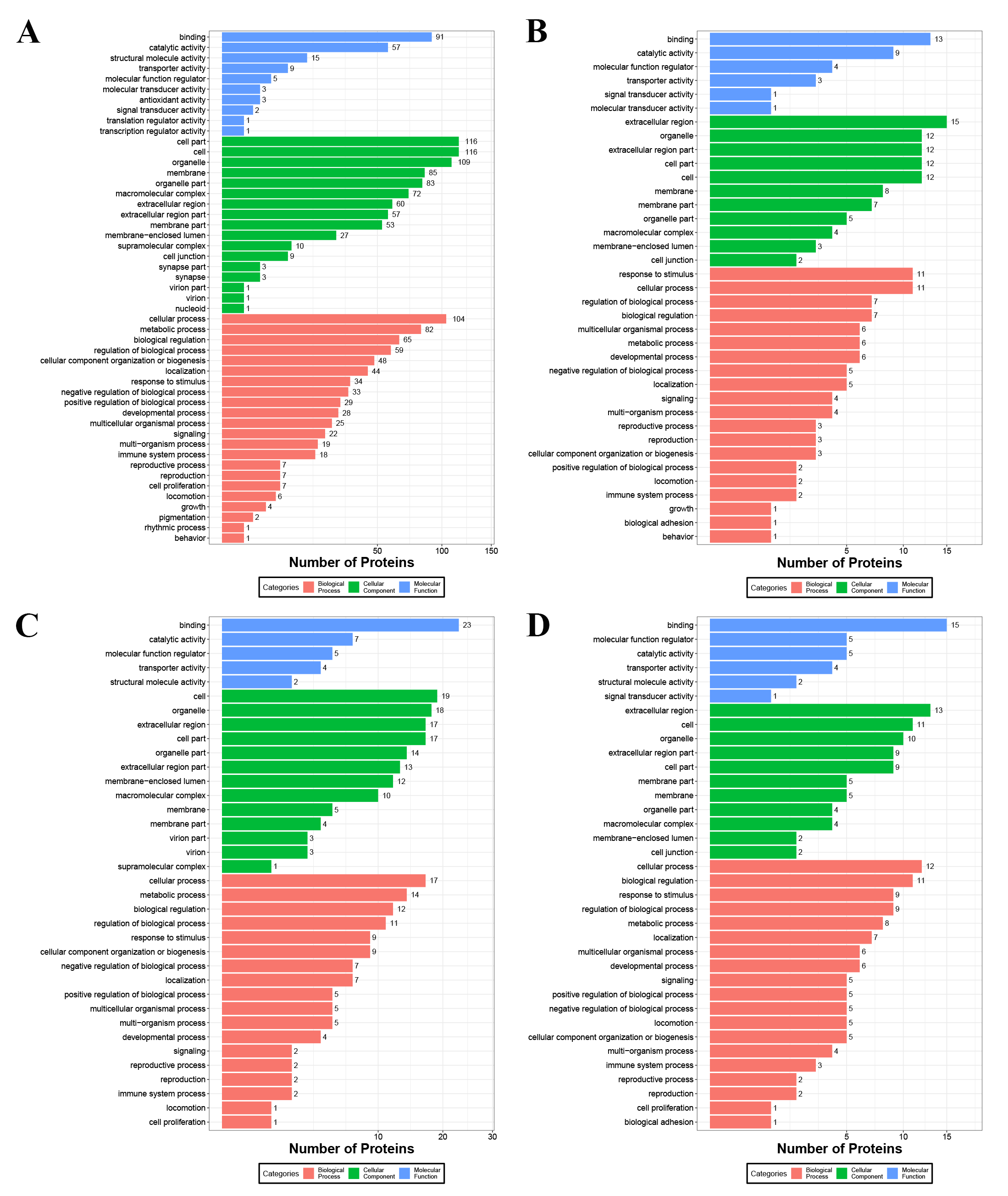

Supplement: Supplementary file 1 [file foods-11-02705-s001.zip › Figure. S1.tif]

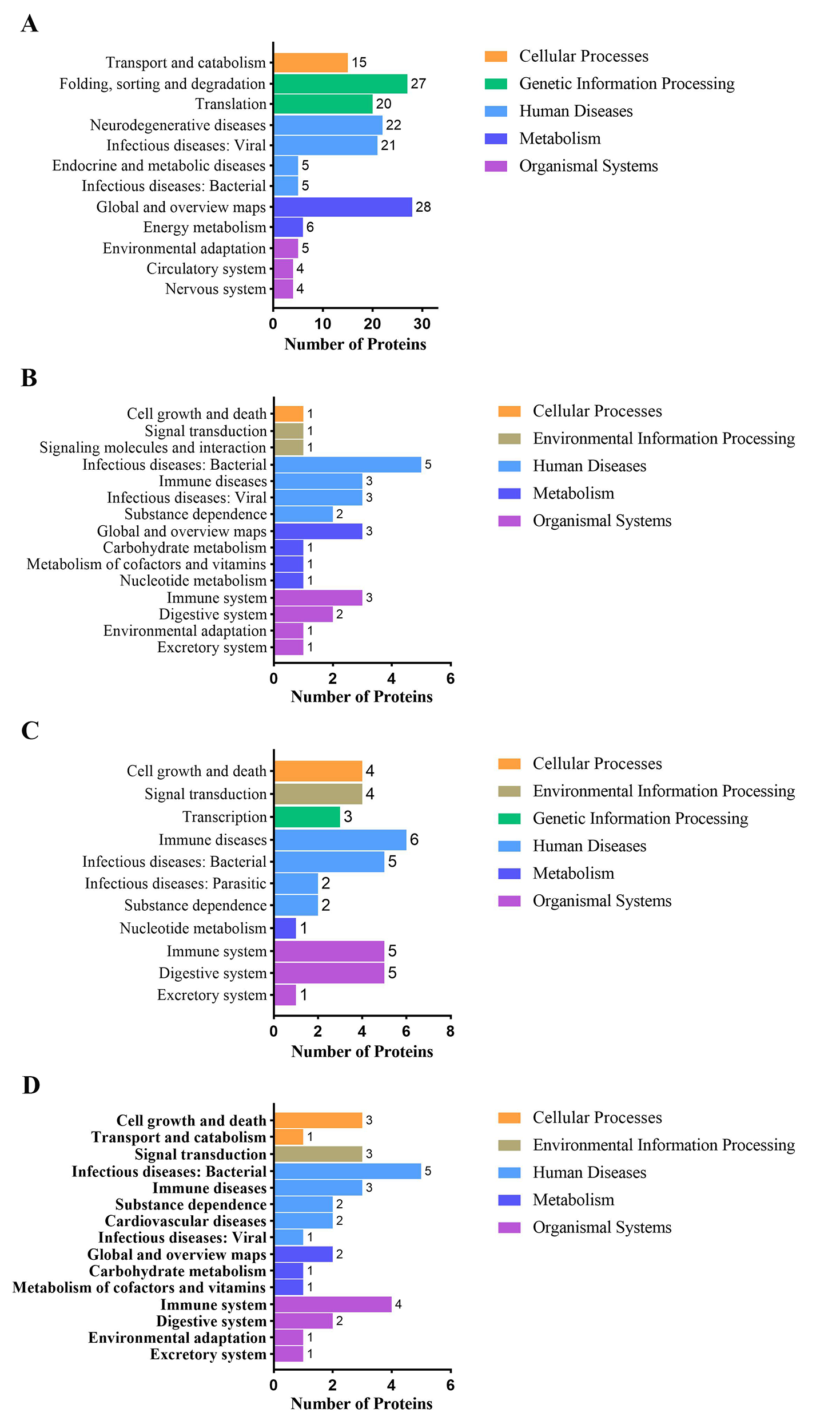

Supplement: Supplementary file 1 [file foods-11-02705-s001.zip › Figure. S2.tif]

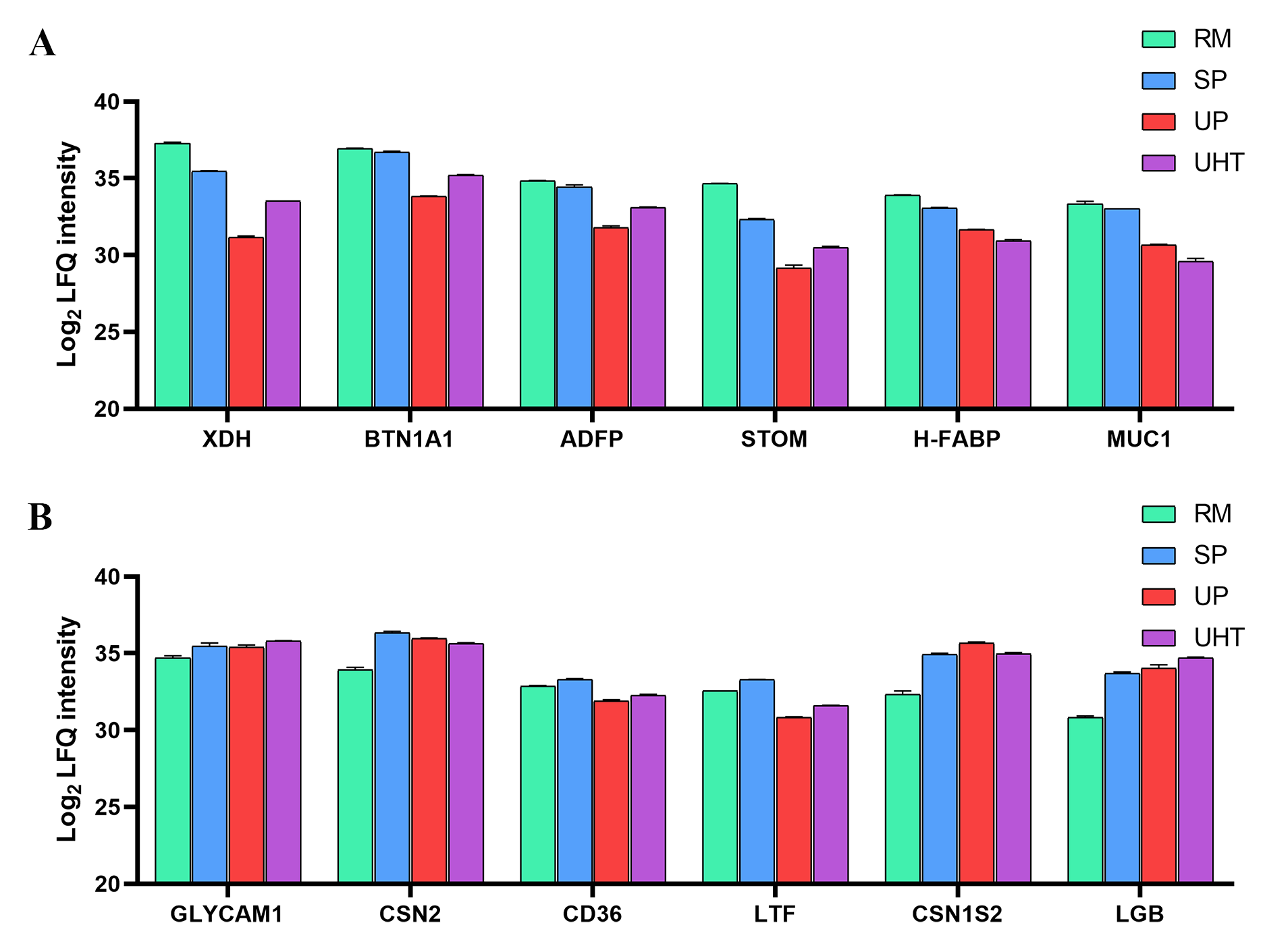

Supplement: Supplementary file 1 [file foods-11-02705-s001.zip › Figure. S3.tif]
